# Implementation mapping to plan for a hybrid trial testing the effectiveness and implementation of a behavioral intervention for HIV medication adherence and care retention

**DOI:** 10.3389/fpubh.2022.872746

**Published:** 2022-08-02

**Authors:** Katelin Hoskins, Amanda L. Sanchez, Carlin Hoffacker, Florence Momplaisir, Robert Gross, Kathleen A. Brady, Amy R. Pettit, Kelly Zentgraf, Chynna Mills, DeAuj'Zhane Coley, Rinad S. Beidas

**Affiliations:** ^1^Penn Medicine Nudge Unit, University of Pennsylvania Health System, Philadelphia, PA, United States; ^2^Department of Psychiatry, Perelman School of Medicine, University of Pennsylvania, Philadelphia, PA, United States; ^3^Penn Implementation Science Center at the Leonard Davis Institute of Health Economics (PISCE@LDI), University of Pennsylvania, Philadelphia, PA, United States; ^4^Department of Counseling and Educational Psychology, Indiana University, Bloomington, IN, United States; ^5^Department of Medicine (Infectious Diseases), Perelman School of Medicine, University of Pennsylvania, Philadelphia, PA, United States; ^6^Center for Clinical Epidemiology and Biostatistics, Perelman School of Medicine, University of Pennsylvania, Philadelphia, PA, United States; ^7^AIDS Activities Coordinating Office, Philadelphia Department of Public Health, Philadelphia, PA, United States; ^8^Independent Consultant, Boston, MA, United States; ^9^Department of Medical Ethics and Health Policy, Perelman School of Medicine, University of Pennsylvania, Philadelphia, PA, United States; ^10^Department of Medicine, Perelman School of Medicine, University of Pennsylvania, Philadelphia, PA, United States; ^11^Center for Health Incentives and Behavioral Economics (CHIBE), University of Pennsylvania, Philadelphia, PA, United States

**Keywords:** implementation science, HIV - human immunodeficiency virus, implementation mapping, health equity (MeSH), stakeholder engagement

## Abstract

**Background:**

Implementation mapping is a systematic, collaborative, and contextually-attentive method for developing implementation strategies. As an exemplar, we applied this method to strategy development for Managed Problem Solving Plus (MAPS+), an adapted evidence-based intervention for HIV medication adherence and care retention that will be delivered by community health workers and tested in an upcoming trial.

**Methods:**

In Step 1: Conduct Needs Assessment, we interviewed 31 stakeholders to identify determinants of MAPS+ implementation in 13 clinics serving people with HIV in Philadelphia County. In Step 2: Develop Logic Model, we used these determinants as inputs for a working logic model guided by the Consolidated Framework for Implementation Research. In Step 3: Operationalize Implementation Strategies, our team held a virtual stakeholder meeting to confirm determinants. We synthesized stakeholder feedback, then identified implementation strategies that conceptually matched to determinants using the Expert Recommendations for Implementing Change taxonomy. Next, we operationalized implementation strategies with specific examples for clinic settings. We linked strategies to behavior change theories to allow for a mechanistic understanding. We then held a second virtual stakeholder meeting to present the implementation menu for feedback and glean generalizable insights for how these strategies could be operationalized in each stakeholder's clinic. In Step 4: Protocolize Strategies, we incorporated stakeholder feedback and finalized the implementation strategy menu.

**Findings:**

Implementation mapping produced a menu of 39 strategies including *revise professional roles, identify and prepare champions, use warm handoffs*, and *change record systems*. The process of implementation mapping generated key challenges for implementation strategy development: lack of implementation strategies targeting the outer setting (i.e., sociopolitical context); tension between a one-size-fits-all and individualized approach for all clinics; conceptual confusion between facilitators and strategies; and challenges in translating the implementation science lexicon for partners.

**Implications:**

This case exemplar advances both MAPS+ implementation and implementation science methods by furthering our understanding of the use of implementation mapping to develop strategies that enhance uptake of evidence-based interventions. The implementation menu will inform MAPS+ deployment across Philadelphia in an upcoming hybrid trial. We will carry out Step 5: Test Strategies to test the effectiveness and implementation of MAPS+.

## Introduction

The primary aim of this paper is to highlight our use of implementation mapping as a systematic, collaborative, and contextually attentive method for developing implementation strategies (1). Implementation mapping identifies context-specific determinants and generates stakeholder-informed implementation strategies, with an eye toward mechanisms (1–3). In this case exemplar detailing our application of implementation mapping in planning for a hybrid type 2 effectiveness-implementation trial, the evidence-based practice (EBP) of interest is MAPS+ and the setting of interest is 13 Ryan White-funded HIV clinics serving people with HIV (PWH) across Philadelphia, Pennsylvania.

### Care gap

Despite steady declines in recent cases, Philadelphia is one of 48 counties in the United States with the highest number of new HIV diagnoses (4). In 2019, new diagnoses were mostly concentrated among people identifying as non-Hispanic Black (64%), people assigned male at birth (76%), and young adults aged 30–39 years old (26%) (5). In 2019, individuals not retained in care accounted for 36% of HIV transmissions, and individuals not virally suppressed but retained in care accounted for 25% of HIV transmissions (6). Notably, Philadelphia is the poorest of the largest U.S. cities, with 23% of residents living in poverty (7). The Ryan White HIV/AIDS Program provides federal grants at the local level to provide care and services for low-income PWH who do not have sufficient health coverage or financial resources (8).

### Evidence-based practice of interest

Managed Problem Solving (MAPS) is an EBP with long-term impact on viral suppression in PWH (9). MAPS consists of four individual-level sessions during the first 3 months of treatment, reinforced by ongoing telephone calls during the 1-year intervention period. The interventionist and participant work together to solve specific adherence barriers using the Problem Solving framework, with an emphasis on small and achievable goals (9, 10). Solutions are tailored toward the specific needs of the participant, empowering them to manage their health. A randomized clinical trial examining MAPS as delivered by college graduate-level interventionists vs. usual care in Philadelphia found that the intervention significantly increased adherence and viral suppression in both treatment-naïve and treatment-experienced patients up to 1 year following MAPS initiation (9). MAPS has been endorsed by the Centers for Disease Control and Prevention as an EBP that improves viral suppression (11); however, as is the case for many EBPs, adoption has been low. Through conversations with the Philadelphia Department of Public Health and HIV clinic directors, our research team learned that MAPS requires adaptation, specifically a need to ensure it can be delivered by non-medical specialists and has an added focus on care retention.

MAPS has been systematically adapted in two key ways to prime the intervention for implementation with the same target population and using the same clinical context as the original trial. First, the delivery system was changed to utilize community health workers (CHWs) instead of personnel with college degrees. Limited staffing in resource-stretched settings has contributed to low adoption. CHWs' inclusion addresses the fact that many health professionals, including medical case managers, do not have the time to offer the intervention within their current responsibilities. Moreover, CHWs function as “trusted liaisons” between health care systems and communities because they often share similar backgrounds as the patients they serve (12). Second, a focus on retention in care was added. In Philadelphia, the greatest barrier to ending the HIV epidemic is poor retention in care among people who are not virally suppressed. The MAPS adaptation process included editing the original MAPS manual to ensure plain language explanations of medical information, providing updated material on adherence supports, and adding material specific to care retention (e.g., explaining the value of regular HIV visits) and problem-solving strategies to address barriers to attendance. The intervention has been renamed MAPS+ to reflect these adaptations. MAPS+ is a valuable tool in service of achieving Ending the HIV Epidemic goals by 2030 (4).

### Hybrid type 2 effectiveness-implementation trial planning

MAPS+ will be tested in an upcoming hybrid type 2 effectiveness-implementation trial in 13 Ryan-White funded clinics in Philadelphia County. Hybrid trials test both clinical effectiveness of interventions and implementation strategies (13), which are the approaches used to increase the adoption, implementation, and sustainment of EBPs (14). In other words, these methods and techniques are the “how” of implementation (14). Strategies are selected to target specific implementation determinants (i.e., barriers and facilitators). For the hybrid trial, we collaboratively identified three primary, multifaceted implementation strategies informed by our conversations with local stakeholders: (1) task-shifting (i.e., redistribution of tasks among health workforce teams from highly qualified health workers to CHWs with less formal training); (2) initial training and ongoing support for CHWs; and (3) integration of the CHW within the clinical team. Examples of integration include developing structures to support information-sharing among the CHW and clinical team members, defining the CHW role and standard work procedures, and having the CHW accompany patients who they serve to their medical appointments. As part of this trial planning, our team also engaged in implementation mapping to elucidate additional implementation strategies that might be needed in collaboration with key partners.

### Implementation mapping to develop implementation strategies

Implementation mapping harnesses insights from both implementation science and intervention mapping (1). It is an approach to implementation strategy development and selection that directly addresses calls to design strategies more systematically, bridging conceptual gaps between determinant identification and strategy selection. As originally described by Fernandez et al. (1), implementation mapping identifies specific, iterative tasks for planners to ensure that attention is paid to all implementers (i.e., individuals putting an intervention into practice), determinants, outcomes, and goals. The approach promotes implementation strategy selection that is shaped by theory and evidence, while also centering the voice of stakeholders and focusing on the mechanisms through which strategies achieve targeted outcomes (1). Selecting strategies to support a change effort is complex, as contextual differences across patient-, provider-, organization-, and system-levels generate variation in implementation (15). As such, the effectiveness of implementation strategies is not context-agnostic (2). Properly selecting strategies to match the multilevel determinants that may enable or hinder implementation is critical, and yet, the methodology of how to do so is underdeveloped. Furthermore, when strategies are developed through atheoretical, haphazard, or non-participatory approaches, it is more difficult to understand mechanisms, that is, the processes by which strategies generate effects on the specified implementation outcomes. Ultimately, care delivery should be informed by theory and stakeholder input (1–3).

Although the principal investigators (RSB, FM, RG) pre-selected three primary implementation strategies for the hybrid trial based on our preliminary understanding of key determinants, we elected to also use implementation mapping to obtain a more nuanced understanding of multilevel context, with an eye to the structural and systemic factors (e.g., power and resource allocation) that likely influence equitable implementation of MAPS+ in Philadelphia. In addition, the strategies identified for the hybrid trial were conceptually broad, and we aimed to enrich our understanding and increase the precision of our strategies in collaboration with clinic stakeholders. Lastly, research suggests that organizations often need to deploy multiple implementation strategies in order to successfully implement an EBP (16–18). In the real-world context of our trial, we sought to further develop auxiliary strategies and track their use prospectively.

Modeled after Fernandez et al.'s (1) approach, our implementation mapping process involved five key steps: (1) Conduct Needs Assessment, (2) Develop Logic Model based on inputs from assessing context, (3) Operationalize Implementation Strategies, (4) Protocolize Strategies, and (5) Test Strategies. Implementation mapping contributed to the development of a detailed implementation blueprint to enhance the three pre-selected implementation strategies and maximize MAPS+ reach, fidelity, and clinical effectiveness. This blueprint will support widespread MAPS+ deployment and scale-up. The work presented here represents Steps 1–4; Step 5 is the hybrid trial. We describe our methods and resulting output as an exemplar of how to design implementation strategies systematically and collaboratively with stakeholders.

## Methods and findings

**Figure 1:**
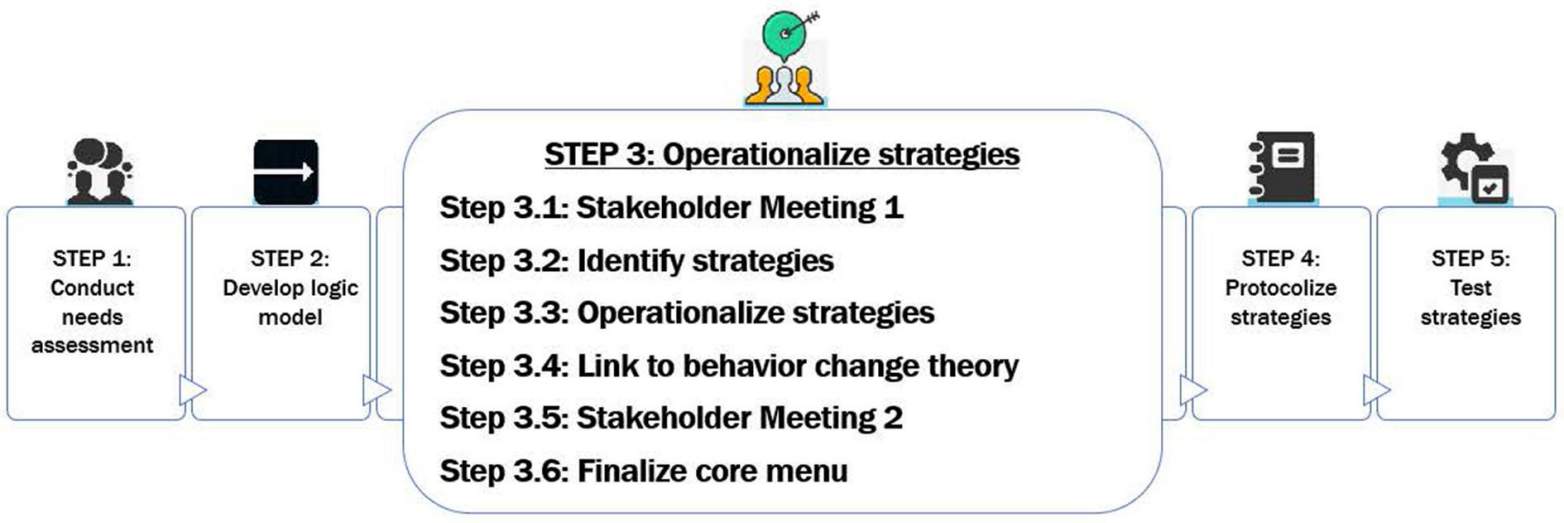
Implementation mapping process.

First, we conducted a needs assessment with stakeholders across 13 clinics serving PWH to understand contextual factors and expected determinants of MAPS+ implementation. Second, we developed a logic model organized by the Consolidated Framework for Implementation Research (CFIR) to conceptually ground our process (19, 20). Third, we operationalized implementation strategies. To do so, we held two stakeholder meetings, mapped strategies to determinants using the empirical dataset and Expert Recommendations for Implementing Change (ERIC) taxonomy (21), generated specific operationalizations, and linked to theory. Fourth, we protocolized the resulting strategies in an implementation menu. We provide a detailed description of our process below and a summary is provided in Figure 1.

### Step 1: Conduct needs assessment

**Figure 2:**
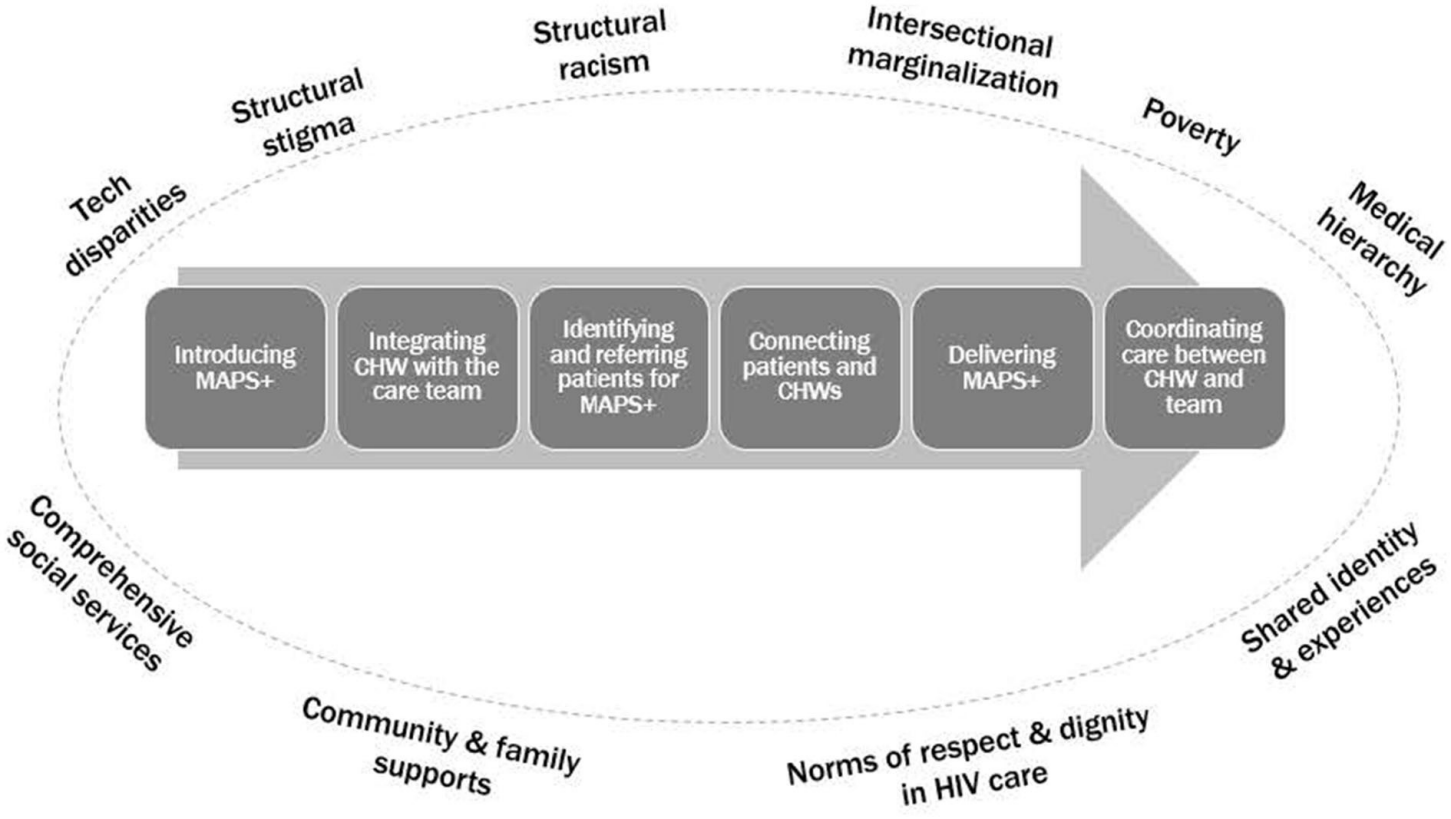
MAPS+ Implementation Pathway from (22).

In order to assess the context for our setting of interest, we completed semi-structured stakeholder interviews (*N* = 31) guided by the CFIR (19) across 13 Ryan White-funded clinics serving PWH in Philadelphia County (22). Our goal was to identify perceived determinants of MAPS+ delivery by CHWs to serve as inputs into the implementation mapping process. Stakeholders included prescribing clinicians (*n* = 6), non-prescribing clinical team members (*n* = 4), clinic administrators (*n* = 7), and policymakers (*n* = 4) from the Philadelphia Department of Public Health. Two research team members (ALS, KH) analyzed these interviews using rapid analytic techniques (23). We used structured interview summaries to populate matrices that aided data organization and pattern identification across stakeholder groups. We then organized determinants by main categories along a MAPS+ Implementation Pathway, which reflected the sequential process of implementing MAPS+ within each clinic (Figure 2). The categories in the pathway included: (1) Introducing MAPS+ to Clinics, (2) Integrating CHW with the Team, (3) Identifying and Referring Patients for MAPS+, (4) Connecting Patients and CHWs, (5) Delivering MAPS+, and (6) Coordinating Care Between CHW and the Team. This process has been described in detail previously (22).

In the Introducing MAPS+ to Clinics category, key determinants included *leadership and staff buy-in*, plus *team expectations for CHW-delivered MAPS*+, meaning expectations about both the CHW role and the purpose of the MAPS+ intervention. The determinants *CHW as core team member, CHW presence on-site, physical space constraints*, and *workflow and role clarity across the team* were important for Integrating CHW with the Team. Specific to the Identifying and Referring Patients for MAPS+ category, we learned that the *structure of existing identification and referral processes* (e.g., data-generated lists) was a key determinant to ensuring that eligible patients were reached. In the category Connecting Patients and CHWs, *CHW availability and scheduling* (and thus accessibility for patients and clinic team members) was key, as were *the initial contact between the CHW and patient*, and *CHW characteristics* (e.g., demographics, experiences, attitudes, skills). *MAPS*+ *characteristics and flexibility* were key determinants in Delivering MAPS+. *Care coordination* and *CHW knowledge of cross-clinic processes* (given that CHWs may work in multiple clinic settings) comprised the Coordinating Care Between the CHW and the Team category (22).

Lastly, we noted factors within the Outer Setting (which includes “the economic, political, and social context within which an organization resides,” (19) that perpetuate inequities, such as structural and systemic racism, intersectional marginalization, structural stigma, and poverty. Structural assets included norms of respect and dignity in HIV care, shared identity and experiences, community and family support, and comprehensive social services (22). Within our analysis, explicitly situating determinants within the broader sociopolitical context of MAPS+ implementation heightened our attention to the complex, historical, and ongoing factors that shape HIV care delivery. Throughout implementation mapping, we anchored on these findings to ensure that implementation strategies were selected through an equity lens, consistent with growing calls to address health equity within implementation science (24–26). The needs assessment findings alerted us to key determinants beyond those associated with the three primary strategies selected for the trial (i.e., *workflow and role clarity*).

### Step 2: Develop logic model

**Figure 3:**
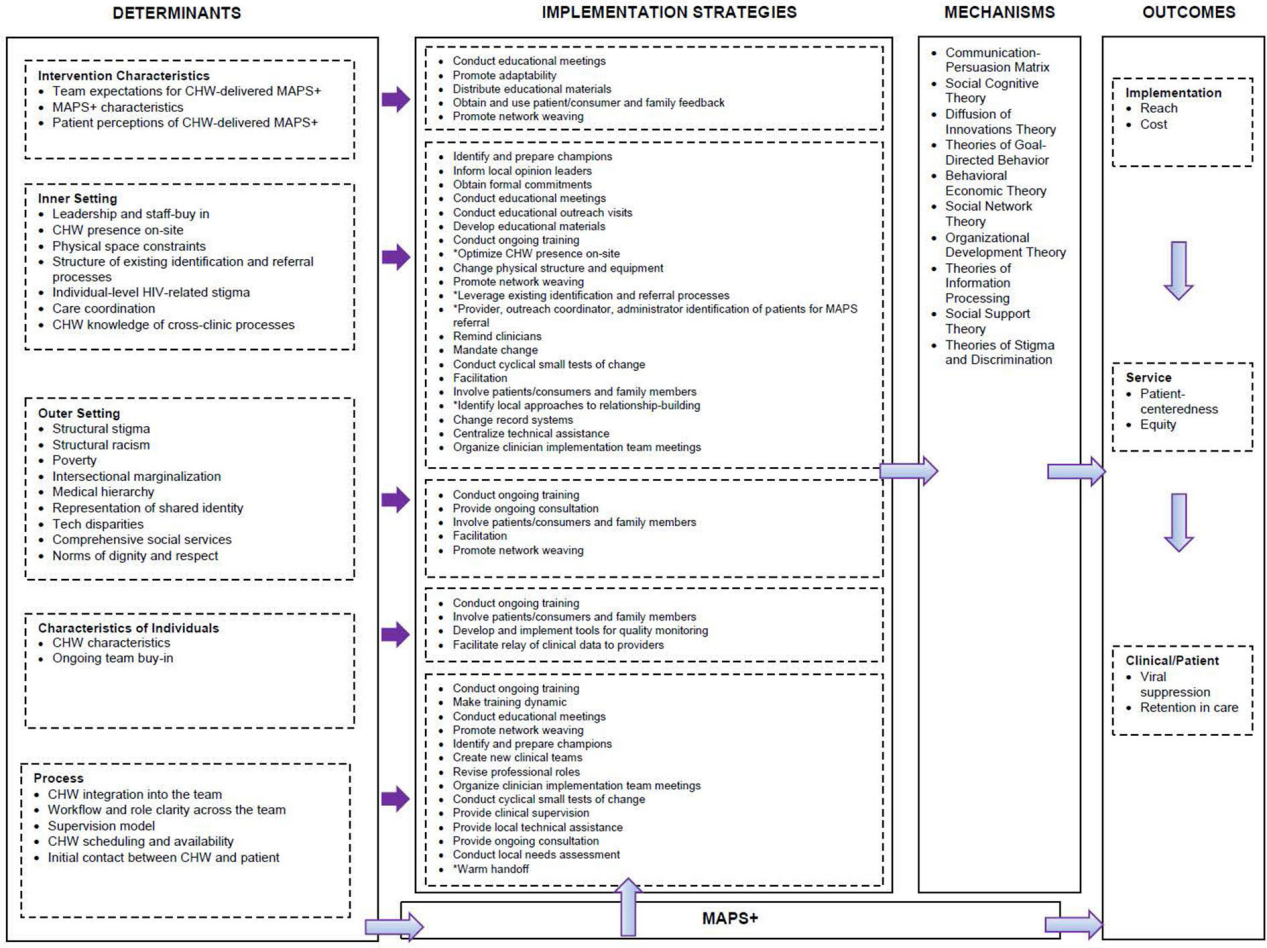
Logic Model filled in by research team iteratively in lead-up to Stakeholder Meeting 2.

We used these determinants as key inputs into a working logic model (Figure 3). The model was organized by ecological levels aligned with the CFIR, specifically intervention characteristics, inner setting, outer setting, characteristics of individuals, and process domains. We modified the Smith et al. (20). Implementation Research Logic Model to increase clarity in the link between each specific CFIR level and relevant strategies and allow for better visualization of which strategies were relevant for each domain and which were applicable across multiple domains (e.g., both inner setting and process). This adapted model served as a conceptually-grounded organizational tool throughout our implementation mapping process (20).

### Step 3: Operationalize implementation strategies

#### Step 3.1: First stakeholder meeting

We held a 90-min virtual stakeholder meeting in May 2021 to present preliminary findings specific to identified determinants, confirm our interpretations, and center the voices of stakeholders. Our research team originally planned for an in-person meeting but pivoted to an online format given COVID-19 mitigation measures. We aimed for representation across a variety of stakeholder groups and clinic settings. To identify participants for the meeting, we collaborated with clinic leadership and contacted potential attendees by email. Eleven stakeholders from 10 different clinics attended, representing prescribing clinician (*n* = 3), medical case manager (*n* = 3), administrator (*n* = 4), and behavioral health consultant (*n* = 1) stakeholder groups. The initial portion of the meeting involved providing an overview of the project, key goals of implementation mapping, and the details of the MAPS+ intervention approach. We then described determinants as categorized by the MAPS+ Implementation Pathway. While we provided a visual of the logic model for “big picture” overview of implementation mapping (Appendix A), we elected to use the pathway as a grounding reference to increase the accessibility of the content for the clinically oriented stakeholders.

Attendees were divided into three breakout groups along with two research team facilitators to support each discussion. Each core project team member (ALS, CH, KH) was paired with a principal investigator (RSB, FM, RG) for the hybrid trial to ensure additional technical expertise related to MAPS+, implementation science, and the upcoming trial. Facilitators all had extensive immersion in the project and were attuned to timing and flow. To support the discussion, facilitators used a guide with suggested discussion points to clarify and confirm our research team's interpretations of determinants. For example, for the determinant *workflow and role clarity across the team* within the Integrating CHW with the Team category, the discussion prompt read as follows: “We heard that it's important for the CHW to have a clearly defined role and to understand specific roles across the multidisciplinary team. Can you tell us more about how to support role clarity for the CHW and for members of the team? What are ways that you have clarified roles for team members in positions that may overlap?” Facilitators and research assistants were provided with a note-taking template to capture detailed feedback. Given the breadth of determinants, each group focused on reviewing one or two categories along the pathway (e.g., Integrating CHW with the Team and Identifying and Referring Patients for MAPS+) to ensure that all categories were discussed. We also encouraged discussion of structural determinants (e.g., poverty) to enhance our understanding of the outer setting.

Following this first stakeholder meeting, the project lead (KH) synthesized the facilitators' meeting notes into a comprehensive document organized along the MAPS+ Implementation Pathway and then the investigative team debriefed. Within the Integrating CHW with the Team category, we learned that clear and consistent messaging related to MAPS+ implementation was critical for both staff and patient buy-in. Education on MAPS+ and the CHW role needed to be upfront with ongoing reinforcement to ensure understanding of the mission. Stakeholders reinforced that CHW role clarity and team cohesion–which included building trust with the new CHW team member–were essential. In addition, CHWs needed to feel valued by the local clinic community. In the Identifying and Referring Patients for MAPS+ category, stakeholders highlighted that staff knowledge about CHW-delivered MAPS+ is key for identification and referral of eligible patients. We also learned that each clinic had structured team meetings and processes to review adherence-related issues, but the timing and structure varied across clinics.

In the Connecting Patients and CHWs category, stakeholders emphasized the importance of CHW availability to patients, including in the evenings and via text message communication. Stakeholders described warm handoff processes in their own clinics. They emphasized the importance of a staff member introducing the CHW to the patient in order to review goals, increase comfort, and build trust. For Delivering MAPS+, stakeholders emphasized delivery flexibility in terms of schedules, setting (office or community), and format (in-person or video platform). To mitigate potential perceptions of burden by patients, they advised framing MAPS+ as an extra support to help patients achieve successful adherence and retention.

In Coordinating Care Between CHW and Team, stakeholders expressed consensus on the importance of clear communication and care coordination. They had contrasting views on the value of communication within the electronic health record (EHR). Some characterized the EHR as an important tool, whereas others noted that providers would not read detailed notes given time scarcity. Alternative communication approaches included brief written treatment plans or HIPAA-secure group texting with action items. Despite clinics having distinct approaches to information-sharing, stakeholders uniformly valued efficiency and accountability. In terms of Outer Setting structural determinants, stakeholders echoed findings from the needs assessment, indicating that unstable housing, inconsistent phone access, limited transportation, and untreated severe mental illness were all major challenges.

Overall, the meeting output confirmed that our approach appropriately reflected stakeholder perspectives and we gleaned new insights to guide implementation strategy selection. We added the category Introducing MAPS+ to the Clinic to the beginning of our implementation pathway to indicate that *leadership and staff buy-in for CHW-delivered MAPS*+ and *team expectations for CHW-delivered MAPS*+ were determinants highly relevant to stakeholders for pre-implementation; this category is described in the publication referenced in Step 1 (22).

#### Step 3.2: Identifying implementation strategies

Given consensus that the findings generated from the interviews were consistent with stakeholder perspectives, the next step was to identify potential implementation strategies that were conceptually matched to determinants. The interview dataset was then used to generate definitions for determinants, pull illustrative examples of determinants, and identify potential implementation strategies voiced by stakeholders (see Appendix B for template). Two research team members (ALS, KH) then independently mapped the determinants onto implementation strategies listed in the refined compilation of implementation strategies from the ERIC taxonomy (21). The documents were merged and reviewed before and during a virtual meeting. In the presence of disagreement, each team member provided rationale, and consensus was reached through productive discussion. After agreeing on key strategies, the project's principal investigator (RSB), an implementation scientist, reviewed the list as an additional confirmatory step. Next, we defined implementation strategies using the refined compilation (Table 1).

**Table 1:** Example of identified determinant, strategies, definitions, operationalizations, and relevant theory per the implementation menu (Step 4 output).

**Determinant**	**Implementation strategies**	**Implementation strategy definitions**	**Implementation strategy operationalizations**	**Relevant theory**
***MAPS+** **Implementation pathway: Introducing MAPS to the clinic***				
**Leadership and staff buy-in for CHW-delivered MAPS+** Definition: Clinic leadership and staff agreement and support for CHW-delivered MAPS+ CFIR: Inner setting- leadership and staff engagement Interview data examples: • Leadership may be resistant to EBP or resistant to change, concern that leadership/providers won't want to buy-in because they are busy and burned out. (Medical Case Manager) • A major facilitator will be getting buy-in from leaders. (Behavioral Health Consultant)	Identify and prepare champions Inform local opinion leaders Obtain formal commitments	*Identify and prepare champions*: identify and prepare individuals who dedicate themselves to supporting, marketing, and driving through an implementation, overcoming indifference or resistance that the intervention may provoke in an organization. *Inform local opinion leaders*: Inform providers identified by colleagues as opinion leaders or “educationally influential” about the clinical innovation in the hopes that they will influence colleagues to adopt it. *Obtain formal commitments*: obtain written commitments from key partners that state what they will do to implement the intervention.	*Identify and prepare champions* • Identify and engage administrators and prescribing clinicians (who are key for referrals) at each clinic site who will commit to successful MAPS+ implementation and support the process across the broader team. *Inform local opinion leaders* • Identify and engage with opinion leaders (may not be administrators or prescribers) to support MAPS+ adoption and sustainment. Frame MAPS+ as an intervention that will add value for both the organization and patients. *Obtain formal commitments* • Identify key asks of implementation partners and obtain written commitments.	*Identify and prepare champions*: Communication-Persuasion Matrix, Social Cognitive Theory, Diffusion of Innovations Theory *Inform local opinion leaders:* Communication-Persuasion Matrix, Diffusion of Innovations Theory *Obtain formal commitments*: Theories of Goal-Directed Behavior, Behavioral Economic Theory

As a check that relevant strategies were not overlooked, we used the CFIR-ERIC Implementation Strategy Matching Tool (27) as an additional guide. After CFIR constructs are entered, the matching tool outputs a summary worksheet with a list of implementation strategies for consideration and prioritizes them based on the percentage of experts who endorsed a strategy as being a “top seven” strategy for the particular barrier (27). To use the matching tool, we mapped determinants to CFIR constructs. For example, the determinant *leadership and staff buy-in for CHW-delivered MAPS*+ aligned with the construct “leadership and staff engagement” within the CFIR inner setting domain. The constructs were then entered into the matching tool. We reviewed the strategies generated by the tool that indicated ≥25% expert endorsement as a top strategy for each barrier (28). We cross-checked these with our identified strategies. Of note, not all of the MAPS+ determinants mapped onto a CFIR construct, particularly determinants related to the sociopolitical context (e.g., medical hierarchy, intersectional marginalization, norms of dignity and respect).

In the process of cross-checking, we scrutinized our determinants and implementation strategies more closely and noted that a few of our facilitators could also be interpreted as implementation strategies. We went back to the original determinants list for reevaluation; using the empirical data, we inferred the determinants driving the miscategorized implementation strategies. For example, the original facilitator of *clinic-level consultation and supervision for the CHW* was actually a more detailed version of the ERIC strategy *clinical supervision* and mapped to the inferred determinant *supervision model* (barrier or facilitator). We also added a new category, Sustaining MAPS+ Implementation, to our implementation pathway after inferring the determinant *ongoing team buy-in* behind the previously identified facilitator *dissemination of effectiveness and outcomes*. The *dissemination* facilitator was a version of the ERIC strategy *develop and implement tools for quality monitoring*, which was defined as “sharing MAPS+ positive outcome data with clinical team by CHW to promote ongoing buy-in.”

#### Step 3.3: Operationalizing implementation strategies

After updating the determinants and implementation strategies, we further operationalized each strategy for clinics serving PWH with several examples generated from our immersion in the data and knowledge of the MAPS+ intervention (Table 1). For example, the strategy *remind clinicians* was operationalized as “bake time into established meetings to review automated (i.e., data generated) referrals as an engagement reminder.” Similarly, *warm handoff* was operationalized as “enact MAPS+ referral in front of/with the patient, in which a team member with an established patient relationship connects the patient to the CHW, explaining why the CHW can address adherence challenges and emphasizing the CHW's competence.” Operationalized strategies were detailed and context-specific.

#### Step 3.4: Linking to theory

The project lead (KH) then linked strategies to relevant theories of behavior change with an emphasis on causal theories to provide a mechanistic understanding of their function. Causal theories included social cognitive theory (29, 30), organizational development theory (30, 31), social network theory (30, 32), and diffusion of innovations theory (30, 33), among others. For example, diffusion of innovations theory explicates the differential rates of intervention adoption across a social system, and interpersonal channels are important for facilitating adoption (30). This theory explains how the implementation strategy *identify and promote champions* works in context; champions are key change agents who support implementation across the organization. We updated this content in the logic model.

Our draft implementation menu included 34 strategies. Of note, six of these strategies were derived directly from the interview data rather than the ERIC compilation, meaning that stakeholders articulated the strategies themselves: *optimize CHW presence on-site*; *provider, outreach coordinator, administrator identification of patients for MAPS*+ *referral*; *identify local approaches to relationship-building; leverage existing identification and referral processes; match scheduling to clinic needs*, and *warm handoffs*. As an example of a non-ERIC strategy definition, *identify local approaches to relationship-building* was defined as “identify strategies that clinics use in routine care to build trust and rapport with patients.” Additional ERIC strategies in the menu included *revise professional roles, promote adaptability*, and *change record systems*.

#### Step 3.5: Second stakeholder meeting

We convened a second virtual stakeholder meeting in July 2021 to present the operationalized implementation strategy menu and obtain feedback on (1) how these strategies might be applied in each stakeholder's clinic and (2) which strategies were most important to stakeholders given finite resources. Feasibility and impact were framed as key constructs in evaluating importance (34). As with our first stakeholder meeting, we strove for representation across a variety of stakeholder groups and clinic settings. We aimed also to include individuals who had not attended the first meeting. In addition, we invited policymakers from the Philadelphia Department of Public Health. Prior to the meeting, we emailed attendees a document listing the 34 implementation strategies and definitions to use as a resource during the presentation and discussion (Appendix C).

Eight clinic stakeholders (*n* = 4 administrators, *n* = 2 medical case managers, *n* = 2 non-prescribing clinical team members) and two policymakers attended the second meeting. We asked clinic stakeholders to reflect on the operationalized strategy examples in order to glean insights that might generalize across clinics. To organize the content for our presentation, we grouped strategies into the nine conceptual clusters (e.g., support clinicians, engage consumers, use evaluative and iterative strategies) from Waltz et al.'s (35) concept mapping project. Appendix D provides an example visual from the meeting and Table 2 lists all strategies by cluster. We labeled each conceptual cluster with a one-word summary (e.g., “evaluate”) for parsimony. Within each breakout group, stakeholders reviewed two or three assigned clusters (e.g., Group 1: Relate/Assist/Adapt clusters, Group 2: Educate/Structure clusters, Group 3: Support/Engage/Evaluate clusters). In light of potential power dynamics, the policymakers were assigned to their own group to reduce discomfort or self-censorship by clinic stakeholders. The policymakers focused on macro considerations, such as how the broader context of care for PWH in Philadelphia interplayed with MAPS+ implementation efforts. As in the first stakeholder meeting, each breakout facilitator used a structured guide. For example, questions in the Group 3 Support/Engage/Evaluate clusters included the following: “Do you foresee any specific challenges with revising and shifting clinical roles?” “What do warm handoffs look like in your clinic?” “Are some clinics better resourced with technology support and quality improvement expertise?” “Are positive outcomes celebrated?” Facilitators and research assistants took detailed notes during the discussion.

**Table 2:** Implementation strategies (*N* = 34) grouped by conceptual cluster for Stakeholder Meeting 2.

**Conceptual cluster**	**Implementation strategy**
Develop stakeholder interrelationships (Relate, *n* = 6)	Identify and prepare champions
	Inform local opinion leaders
	Obtain formal commitments
	Promote network weaving
	Organize clinician implementation team meetings
	Identify local approaches to relationship-building
Provide interactive assistance (Assist, *n* = 5)	Facilitation
	Provide clinical supervision
	Provide ongoing consultation
	Provide local technical assistance
	Centralize technical assistance
Adapt and tailor to context (Adapt, *n* = 1) Train and educate stakeholders (Educate, *n* = 6)	Promote adaptability Conduct educational meetings Develop educational materials Distribute educational materials Conduct educational outreach visits Conduct ongoing training Make training dynamic
Change infrastructure (Structure, *n* = 6)	Change physical structure and equipment
	Leverage existing identification and referral processes
	Provider, outreach, coordinator, or administrator identification of patients for MAPS+ referral
	Mandate change
	Match scheduling to clinic needs
	Change record systems
Support clinicians (Support, *n* = 8)	Create new clinical teams
	Revise professional roles
	Optimize CHW presence on-site
	Remind clinicians
	Facilitate relay of clinical data to providers
	Warm handoffs
	Involve patients and family members
	Obtain and use patient and family feedback
Use evaluative and iterative strategies (Evaluate, *n* = 2)	Conduct cyclical small tests of change
	Develop and implement tools for quality monitoring

Following the meeting, the project lead (KH) synthesized the facilitator notes and prepared a report that mapped specific stakeholder feedback to each implementation strategy. The report highlighted key takeaways (e.g., highly salient points that included nuanced feedback) specific to operationalizations of several implementation strategies. The investigative team debriefed and discussed how the takeaways could further inform implementation strategy development. For the strategy *conduct educational meetings*, we learned that stakeholders viewed the meetings as key for MAPS+ implementation launch but felt they must be brief, focused, and tailored for each internal stakeholder group (e.g., prescribing clinicians vs. medical case managers). Stakeholders emphasized the value of *revise professional roles*, highlighting that role clarity is essential. Adding the CHW to the team requires addressing potential duplication of roles that may create burden for patients (e.g., needing to repeat the same component of their medical history to multiple team members). Moreover, good handoffs are tied to a clear understanding of team members' roles. Specific to the strategy *develop and implement tools for quality monitoring*, stakeholders noted that clinic teams receive numerous data-driven reports and that MAPS+ outcomes should be highlighted to increase attention from the team.

We also gleaned five new implementation strategies from this meeting. First, we heard that many clinics have already remediated problems and streamlined processes for other interventions. With this experience, clinic staff planned to identify how the CHW model for MAPS+ delivery can fold into their specific workflows. This information led us to create *leverage existing processes and procedures specific to each clinic* as a distinct strategy. The other four strategies were derived from policymaker input: *communicate feedback on structural barriers back to clinic leadership and Philadelphia Department of Public Health*; *integrate research team into learning collaboratives*; *have research team engage with a collaborative between HIV care and prevention service users and providers*; and *have research team present at community-based organization meeting*. Overall, this second stakeholder meeting yielded concrete input on strategy operationalization as well as consensus on areas to prioritize (e.g., educational meetings).

### Step 4: Protocolize implementation strategies

This feedback was further synthesized with input from the investigative team to finalize a core menu of 39 implementation strategies (Table 3), which aligns with prior research on specifying and reporting implementation strategies that has found a range of 11 to 45 strategies per implementation study (16–18). This core menu, referred to as the team's implementation blueprint, will inform the deployment of MAPS+. The menu is organized by determinants, matched implementation strategies, strategy definitions, strategy operationalizations, and associated theory. The full menu is available in Appendix E.

**Table 3:** Final list of implementation strategies (*N* = 39).

1	Centralize technical assistance
2	Change physical structure and equipment
3	Change record systems
4	*Communicate feedback on structural barriers back to clinic leadership and PDPH
5	Conduct cyclical small tests of change
6	Conduct educational meetings
7	Conduct educational outreach visits
8	Conduct ongoing training
9	Create new clinical teams
10	Develop and implement tools for quality monitoring
11	Develop educational materials
12	Distribute educational materials
13	Facilitate relay of clinical data to providers
14	Facilitation
15	Identify and prepare champions
16	*Identify local approaches to relationship-building
17	Inform local opinion leaders
18	*Integrate research team into learning collaboratives
19	Involve patients/consumers and family members
20	*Leverage existing identification and referral processes
21	*Leverage existing processes and procedures specific to each clinic
22	Make training dynamic
23	Mandate change
24	*Match scheduling to clinic needs
25	Obtain and use patients/consumers and family feedback
26	Obtain formal commitments
27	*Optimize CHW presence on-site
28	Organize clinician implementation team meetings
29	Promote adaptability
30	Promote network weaving
31	Provide clinical supervision
32	Provide local technical assistance
33	Provide ongoing consultation
34	Provider, outreach coordinator, administrator identification of patients for MAPS+ referral
35	Remind clinicians
36	*Research team engagement with a collaborative between HIV care and prevention service users and providers
37	*Research team presentation at community-based organization meeting
38	Revise professional roles
39	*Warm handoffs

*Non-ERIC implementation strategies derived directly from interviews and stakeholder meetings.

## Challenges and lessons learned

In summary, our structured implementation mapping process generated 39 implementation strategies systematically and collaboratively with stakeholders. In Step 1: Conduct Needs Assessment, our analysis of stakeholder interviews yielded contextually-rich insights into the determinants of MAPS+ implementation across clinics in Philadelphia. These empirical data anchored our inquiry; we frequently returned to the interview dataset to clarify, confirm, and center stakeholders' experiences. In Step 2: Develop Logic Model, we linked determinants to CFIR domains and input these determinants into a modification of Smith et al.'s (20), Implementation Research Logic Model. We updated the logic model throughout the course of implementation mapping. In Step 3: Operationalize Implementation Strategies, we held Stakeholder Meeting 1 to confirm determinants (3.1); identified implementation strategies that conceptually matched to determinants from the ERIC compilation and interview dataset (3.2); and operationalized implementation strategies with specific examples (3.3). We then linked strategies to theories of behavior change (3.4) and held Stakeholder Meeting 2 to present the menu for feedback (3.5). In Step 4: Protocolize Strategies, we finalized the core implementation strategy menu. Each element of Steps 3-4 supported scrutiny of each identified strategy to ensure both conceptual and practical relevance for implementation.

Throughout our implementation mapping process, we identified several challenges–lack of implementation strategies targeting outer setting, tension between one-size-fits-all and individualized approach for all clinics, lack of clarity between facilitators and strategies, and challenges in translating the implementation science lexicon to make it relevant for partners–which we reflect on here. First, we noted a scarcity of implementation strategies targeting outer setting. The equity-related determinants (e.g., structural stigma, racism, poverty) highlighted in our needs assessment called for direct attention to the sociopolitical context of implementation. In addition to integrating consideration of outer setting into our stakeholder meetings, the team reviewed the literature. Engaging with theory beyond the realm of implementation science provided traction for understanding the historically-rooted cultural norms and institutional polices that can inhibit opportunities and wellbeing for PWH (36). We found little relevant literature for implementation strategies targeted to these structural determinants. We selected *conduct ongoing training, provide ongoing consultation*, and *involve patients/consumers and family members* as the most relevant strategies from the ERIC taxonomy to address these barriers. These strategies are limited in their application beyond the individual level, which is problematic given that the success of implementation is fundamentally bound by structural constraints enacted by upstream institutional policies, practices, and norms.

Aside from the limitations of equity-informed implementation strategies in the literature, policymaker engagement in our second stakeholder meeting elicited novel system-level strategies that we added to our core menu. Besides *engagement with an existing collaborative of organizations*, policymakers also identified the importance of a mechanism to *communicate feedback* for CHWs to inform clinic leadership and the Philadelphia Department of Public Health on patients' experiences with structural barriers that impede MAPS+ participation. Development of this communication mechanism could enhance implementation in two important ways: (1) institutional investment in the authority and value of CHW knowledge and (2) multilevel problem-solving in direct service of PWH. New models of “flipping the paradigm,” in which CHWs mentor health care system executives, hold promise for cultivating cultural humility and structural competency among agents who wield the most power (12). Methodologically in this project, diverse stakeholder input was essential for generating strategies across all ecological levels. Beyond the scope of this case, increased development and reporting of strategies that target outer setting (i.e., macro) determinants is critical to advancing more equitable implementation, particularly for historically marginalized groups with intersectional barriers.

Second, we observed a tension between a one-size-fits-all and an individualized approach for all clinics. Given the heterogeneity of internal processes across our 13 partner clinics, individual determinants differed across clinics. These differences created considerations for adaptation and tailoring of implementation strategies. Although the same strategies derived from implementation mapping will be used in all clinics (e.g., *conduct educational meetings, develop educational materials*), they may need to be adapted to the local context (37). Strategy adaptations are planned, proactive modifications (38); the strategy might be different in form whereas the function is the same. Function attends to structural and procedural goals (i.e., the core purposes of the strategy), and the form is the operationalization (39). For example, *warm handoffs* serve the function of initiating a transparent transfer of care (40). In front of the patient, the established care team member signals trust and imbues confidence in the new CHW. How clinics plan to implement the form of *warm handoffs* may differ, with individual clinic variation in the handoff initiator (e.g., clinician or case manager) and timing (e.g., in the clinical encounter or during next appointment scheduling) based on workflow.

In contrast to adaptation, strategy tailoring reflects the presence or absence of a strategy based on clinic context. While we have pre-selected strategies based on context and the design of the study (i.e., task-shifting, initial training and ongoing support for CHWs, integration of the CHW within the clinical team), not all auxiliary strategies will be deployed in all clinics. Some strategies may be more germane to certain clinics than others based on context. Other strategies may be ancillary (e.g., *provide local technical assistance, provide ongoing consultation, organize clinician implementation team meetings*). As such, the use of implementation strategies can be tailored to the context-specific factors for each clinic identified during the pre-implementation needs assessment (4). Given the breadth of determinants across clinics, identifying which strategies should be deployed across all clinics (then adapted to context) vs. deployed to specific clinics (tailored to context-specific determinants) is a key consideration for our research team.

A third challenge was lack of clarity between facilitators and strategies. In analyzing the interview data, we had difficulty distinguishing facilitators from implementation strategies with regard to what would make implementation of MAPS+ easier. As described above, in reviewing determinants using the CFIR-ERIC Matching Tool, we identified a few facilitators that appeared to be implementation strategies and required recategorization (e.g., *CHW onboarding and training* was a distinct strategy, not a facilitator). To properly recategorize, we needed to return to the data to infer the determinants behind the articulated strategy. Inferring determinants from stakeholder-proposed solutions is an approach used in prior studies that has helped to identify the factors that may impede implementation of EBPs (41). We found that the mental heuristic of facilitators as “nouns” (extant key factors) and implementation strategies as “verbs” (added key actions) helped our team delineate facilitators from strategies. Overall, we noted challenges specific to limited precision with facilitators and an outstanding question about the extent to which facilitators and strategies may overlap. This ambiguity highlights a need to increase conceptual clarity around enablers of implementation. Our need to return to the data highlights the flexibility and iteration required for implementation mapping.

Lastly, we experienced challenges in translating established implementation science lexicon and taxonomies to our partner stakeholders. We recognized that terminology related to conceptual frameworks, determinants, and implementation strategies (with dense names like “*facilitation*”) did not resonate with our stakeholders, who contributed their own deep, discipline-specific knowledge of HIV care within the city. Moreover, implementation strategy definitions were not always clear, even to our research team (e.g., defining “*network weaving*”). Our stakeholder meeting materials required multiple refinements to improve clarity. As described above, we also constructed a resource document with specific definitions of implementation menu strategies to increase accessibility. In addition, we realized that the MAPS+ Implementation Pathway, which grouped determinants sequentially and served us well in the first meeting, was less useful for presenting implementation strategies. Some strategies (e.g., *identify local opinion leaders*) were associated with determinants in pre-implementation only, whereas other strategies (e.g., *organize clinician implementation team meetings*) were identified across multiple stages. The temporality of implementation strategies–that is, whether the specific strategy was applicable within one implementation stage or across multiple stages–was particularly difficult to convey. Ultimately, organizing strategies by conceptual cluster was an efficient approach that resulted in meaningful output from the second stakeholder meeting.

## Limitations

As only one team member (KH) had completed linkage between strategies and theory, our list represents a preliminary understanding of mechanisms. Use of theory will be further refined in future work. We did not use quantitative measures to obtain rankings of stakeholders' preferences for implementation strategies in Stakeholder Meeting 2. And finally, in Step 4 we elected not to specify implementation strategies per Proctor et al.'s (14) reporting guidance with details about the actor, action, action targets, temporality, dose implementation outcomes addressed. This important work will be carried out in the context of the upcoming trial, described below.

## Future directions

The implementation menu from Step 4 will populate an implementation strategy tracker with strategy specification per reporting guidance (14). The tracker will be updated monthly during the trial. Our implementation blueprint facilitated more comprehensive planning for the trial, and we can now formally and prospectively track what strategies were planned in advance vs. modified in reaction to unanticipated barriers that arose in clinics during implementation (37). We will then describe how and why strategies succeeded (or failed) so they can be replicated or further refined in future implementation efforts (42). Our process thus far has yielded knowledge generalizable to other behaviorally informed EBPs for HIV/AIDS.

Our case exemplar illustrates a systematic process of designing implementation strategies for a broad-scale, multi-site implementation effort. Use of implementation mapping is a unique contribution to the HIV/AIDS research community with great promise for promoting Ending the HIV Epidemic goals and improving outcomes for PWH. The method may be especially valuable for other health domains in which the social context is complex and underexplored through an implementation lens. We see opportunities for further delineation of implementation mapping steps to increase the accessibility of this method for investigators new to implementation science. We also encourage investigators to expand on the solutions we generated specific to the challenges of this case exemplar.

## Data availability statement

The original contributions presented in the study are included in the article/Supplementary materials; further inquiries can be directed to the corresponding author.

## Ethics statement

The studies involving human participants were reviewed and approved by the City of Philadelphia Institutional Review Board. Written informed consent for participation was not required for this study in accordance with national legislation and the institutional requirements.

## Author contributions

KH, ALS, CH, FM, RG, and RSB contributed to the conception and design of the study. RSB served as project director of the supplement award. KH wrote the first draft of the manuscript. All authors contributed to manuscript revision, and read and approved the submitted version.

## Funding

This publication was made possible in part through an Ending the HIV Epidemic Supplement from the Penn Center for AIDS Research (CFAR; P30 045088), the Penn CFAR ISPHERE Scientific Working Group (P30 045088), and Penn Mental Health AIDS Research Center (P30 MH097488). ALS and KH received funding from the National Institute of Mental Health Training Fellowship (T32 MH109433; Mandell/Beidas MPIs).

## Conflict of interest

RSB receives royalties from Oxford University Press, currently provides consultation to United Behavioral Health, and serves on the Clinical and Scientific Advisory Board for Optum Behavioral Health. ARP has consulted on research studies funded by AbbVie, RG serves on the data and safety monitoring board for Pfizer Inc.

The remaining authors declare that the research was conducted in the absence of any commercial or financial relationships that could be construed as a potential conflict of interest.

## Publisher's note

All claims expressed in this article are solely those of the authors and do not necessarily represent those of their affiliated organizations, or those of the publisher, the editors and the reviewers. Any product that may be evaluated in this article, or claim that may be made by its manufacturer, is not guaranteed or endorsed by the publisher.
