# Supplementary material for: Implementation mapping to plan for a hybrid trial testing the effectiveness and implementation of a behavioral intervention for HIV medication adherence and care retention
Source: Front Public Health. 2022 Aug 2;10:872746. doi: 10.3389/fpubh.2022.872746 (PMC9379308; doi:10.3389/fpubh.2022.872746)
Supplement: Supplementary file 1 [file Data_Sheet_1.PDF]

Appendix A. Implementation mapping and logic model overview slide presented to clinic staff at Stakeholder Meeting 1.

Implementation Mapping: Big Picture

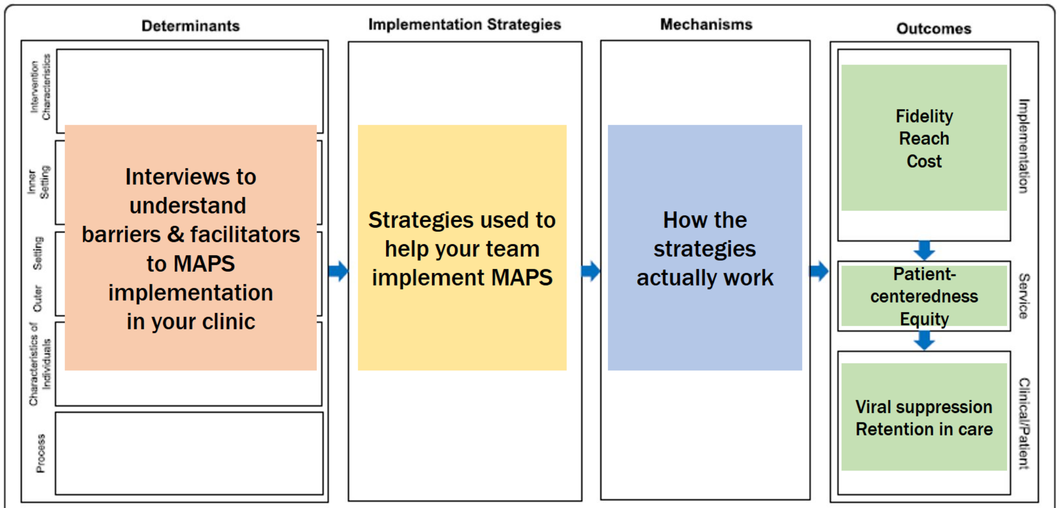

Fig. 1 Implementation Research Logic Model (IRLM) Standard Form. *Notes.* Domain names in the determinants section were drawn from the Consolidated Framework for Implementation Research. The format of the outcomes column is from Proctor et al. 2011

(Smith, Li & Rafferty, 2020)

Appendix B. Template functioning as an organizer and referent for implementation mapping users.

	**Determinant**	**Implementation Strategies**	**Implementation Strategy Definitions**	**Implementation Strategy Operationalizations**	**Relevant Theory**
#[]	**[DETERMINANT NAME]**<br><br>[Determinant Definition]<br><br>[CFIR: domain and construct]	[IMPLEMENTATION STRATEGIES ASSOCIATED WITH DETERMINANT]	[IMPLEMENTATION STRATEGY NAME + DEFINITION FROM ERIC COMPLILATION OR THE EMPIRICAL DATA]	[IMPLEMENTATION STRATEGY NAME + SPECIFIC EXAMPLE FOR USE IN LOCAL CONTEXT]	[THEORY RELEVANT FOR EACH SPECIFIC STRATEGY]

	[Examples from needs assessment]				

Appendix C. Resource with defined implementation strategies for Stakeholder Meeting 2.

Stakeholder Meeting 2: Implementation Strategy Cheat Sheet

Implementation Strategy	Definition
1. Centralize technical assistance	Develop and use a centralized system to deliver technical assistance focused on implementation issues.
2. Change physical structure and equipment	Evaluate current configurations and adapt, as needed, the physical structure and/or equipment (e.g., changing the layout of a room, adding equipment) to best accommodate MAPS.
3. Change record systems	Change records systems to allow better assessment of implementation or clinical outcomes.
4. Conduct cyclical small tests of change	Implement changes in a cyclical fashion using small tests of change before taking changes system-wide. Tests of change benefit from systematic measurement, and results of the tests of change are studied for insights on how to do better. This process continues serially over time, and refinement is added with each cycle.
5. Conduct educational meetings	Hold meetings targeted toward different stakeholder groups (e.g., providers, administrators, other organizational stakeholders, and community, patient/consumer, and family stakeholders) to teach them about MAPS.
6. Conduct educational outreach visits	Have a trained person meet with providers in their practice settings to educate providers about MAPS with the intent of changing the provider's practice.
7. Conduct ongoing training	Plan for and conduct training in MAPS in an ongoing way.
8. Create new clinical teams	Change who serves on the clinical team, adding different disciplines and different skills to make it more likely that MAPS is delivered (or is more successfully delivered).
9. Develop and implement tools for quality monitoring	Develop, test, and introduce into quality-monitoring systems the right input—the appropriate language, protocols, algorithms, standards, and measures (of processes, patient/consumer outcomes, and implementation outcomes) that are often specific to MAPS.
10. Develop educational materials	Develop and format manuals, toolkits, and other supporting materials in ways that make it easier for stakeholders to learn about the innovation and for clinicians to learn how to deliver MAPS.
11. Distribute educational materials	Distribute educational materials (including guidelines, manuals, and toolkits) in person, by mail, and/or electronically.
12. Facilitate relay of clinical data to providers	Provide as close to real-time data as possible about key measures of process/outcomes using integrated modes/channels of communication in a way that promotes use of MAPS.
13. Facilitation	A process of interactive problem solving and support that occurs in a context of a recognized need for improvement and a supportive interpersonal relationship.
14. Identify and prepare champions	Identify and prepare individuals who dedicate themselves to supporting, marketing, and driving through an implementation, overcoming indifference or resistance that MAPS may provoke in an organization.
15. *Identify local approaches to relationship-building	Identify strategies that clinics use in routine care to build trust and rapport with patients.
16. Inform local opinion leaders	Inform providers identified by colleagues as opinion leaders or "educationally influential" about MAPS in the hopes that they will influence colleagues to adopt it.

17.	Involve patients/consumers and family members	Engage or include patients/consumers and families in the implementation effort.
18.	*Leverage existing identification and referral processes	Use of existing clinic processes (i.e., Data to Care, CAREWare, EHR) to identify and refer patients for MAPS.
19.	Make training dynamic	Vary the information delivery methods to cater to different learning styles and work contexts and shape the training in MAPS to be interactive.
20.	Mandate change	Have leadership declare the priority of MAPS and their determination to have it implemented.
21.	*Match scheduling to clinic needs	Collect and analyze data related to the optimal clinic times for CHW scheduling.
22.	Obtain and use patients/consumers and family feedback	Develop strategies to increase patient/consumer and family feedback on the implementation effort.
23.	Obtain formal commitments	Obtain written commitments from key partners that state what they will do to implement MAPS.
24.	*Optimize CHW presence on-site	Identify key times to facilitate CHW presence at the clinic site.
25.	Organize clinician implementation team meetings	Develop and support teams of clinicians who are implementing MAPS and give them protected time to reflect on the implementation effort, share lessons learned, and support one another's learning.
26.	Promote adaptability	Identify the ways MAPS can be tailored to meet local needs and clarify which elements of MAPS must be maintained to preserve fidelity.
27.	Promote network weaving	Identify and build on existing high-quality working relationships and networks within and outside the organization, organizational units, teams, etc. to promote information sharing, collaborative problem-solving, and a shared vision/goal related to implementing MAPS.
28.	Provide clinical supervision	Provide clinicians with ongoing supervision focusing on MAPS. Provide training for clinical supervisors who will supervise clinicians who provide MAPS.
29.	Provide local technical assistance	Develop and use a system to deliver technical assistance focused on implementation issues using local personnel.
30.	Provide ongoing consultation	Provide ongoing consultation with one or more experts in the strategies used to support implementing MAPS.
31.	*Provider, outreach coordinator, administrator identification of patients for MAPS referral	Team member (i.e., provider, outreach coordinator, MCM, administrator) identification of patients for MAPS referral to augment exclusively data-driven processes.
32.	Remind clinicians	Develop reminder systems designed to help clinicians to recall information and/or prompt them to use MAPS.
33.	Revise professional roles	Shift and revise roles among professionals who provide care, and redesign job characteristics.
34.	Warm handoff	Transparent transfer of care between two members of the health care team, where the handoff occurs in front of the patient.

*Specific IS for this project. All other definitions customized from: Powell B et al. (2015). A refined compilation of implementation strategies: results from the Expert Recommendations for Implementation Change (ERIC) project. *Implementation Science*, *10*(21). DOI 10.1186/s13012-015-0209-1

Appendix D. Slide with implementation strategies grouped by conceptual cluster presented to clinic staff and policymakers at Stakeholder Meeting 2.

SUPPORT

- Create new clinical teams
- Revise professional roles
- Optimize CHW presence on-site
- Remind clinicians
- Facilitate relay of clinical data to providers

ENGAGE

- Warm handoffs
- Involve patients and family members
- Obtain and use patient and family member feedback

EVALUATE

- Conduct cyclical small tests of change
- Develop and implement tools for quality monitoring

Appendix E. Full implementation menu.

	Determinant	Implementation Strategies<br>*non-ERIC IS derived directly from interviews/stakeholder data	IS Definitions	IS Potential Operationalization<br>┼derived directly from interviews/stakeholder data	Relevant Theory
Introducing MAPS+ to the Clinic					
1	**Leadership and staff buy-in for CHW-delivered MAPS+**<br>Clinic leadership and staff agreement and support for CHW-delivered MAPS+<br><br>CFIR: Inner setting-leadership and staff engagement<br><br><u>Examples</u>:<br>• Leadership may be resistant to EBP or resistant to change, concern that leadership/providers won't want to buy-in because they are busy and burned out. (Medical Case Manager)<br>• A major facilitator will be getting buy-in from leaders. (Medical Case Manager)<br>• Educating the team on this program ahead of time so that	Identify and prepare champions<br><br>Inform local opinion leaders<br><br>Obtain formal commitments<br><br>Conduct educational meetings<br><br>Develop educational materials	*Identify and prepare champions*: identify and prepare individuals who dedicate themselves to supporting, marketing, and driving through an implementation, overcoming indifference or resistance that the intervention may provoke in an organization.<br><br>*Inform local opinion leaders*: Inform providers identified by colleagues as opinion leaders or "educationally influential" about the clinical innovation in the hopes that they will influence colleagues to adopt it.<br><br>*Obtain formal commitments*: obtain written commitments from key partners that state what they will do to implement the intervention.	*Identify and prepare champions*<br>• ┼Identify and engage administrators and prescribing clinicians (who are key for referrals) who will commit to successful MAPS implementation and supporting the process across the broader team.<br><br>*Inform local opinion leaders*<br>• Identify and engage with opinion leaders within the organization (may not be administrators or prescribers) to support MAPS+ adoption and sustainment.<br>• ┼Frame MAPS+ as an intervention that will add value (as opposed to burden) for both the organization and patients.<br><br>*Obtain formal commitments*<br>• Identify key asks of implementation partners and obtain written commitments.<br>• Leverage social pressure and close intention-action gaps with public	*Identify and prepare champions*: Communication-Persuasion Matrix, Social Cognitive Theory, Diffusion of Innovations Theory<br><br>*Inform local opinion leaders:* Communication-Persuasion Matrix, Diffusion of Innovations Theory<br><br>*Obtain formal commitments*: Theories of Goal-Directed Behavior, Behavioral Economic Theory<br><br>*Conduct educational meetings*: Communication-Persuasion Matrix, Social Cognitive Theory

	they know who this person is and what their function is, get them on board with the program. (Behavioral Health Consultant)		*Conduct educational meetings*: Hold meetings targeted toward different stakeholder groups (e.g., providers, administrators, other organizational stakeholders, and community, patient/consumer, and family stakeholders) to teach them about the clinical innovation.<br><br>*Develop educational materials:* Develop and format manuals, toolkits, and other supporting materials in ways that make it easier for stakeholders to learn about the innovation and for clinicians to learn how to deliver the clinical innovation.	commitments, e.g., online leaderboards, pledges, and/or emails within and across organizations specific to the implementation commitment.<br><br>*Conduct educational meetings:*<br>• Hold educational meetings tailored to each stakeholder group (e.g., prescribing clinicians, MCMs, BHCs) during pre- and early implementation to promote buy-in (and develop competency).<br>• Identify best practices for educating new/incoming team members about CHW-delivered MAPS+.<br><br>*Develop educational materials*<br>• Develop manual and other readily accessible resources (e.g., 1-pagers, visual reminders) specific to MAPS+ intervention. Provide effectiveness data to increase buy-in.<br>• Develop patient-facing educational materials specific to CHW-delivered MAPS+ in accessible format (i.e., attentive literacy, language).	*Develop educational materials*: Social Cognitive Theory
2	**Team expectations for CHW-delivered MAPS+**<br><br>Clear expectations from clinic team about the	Conduct educational meetings	*Conduct educational meetings*: Hold meetings targeted toward different stakeholder groups (e.g., providers, administrators,	*Conduct educational meetings*<br>• Hold educational meetings tailored to each stakeholder group (e.g., prescribing clinicians, MCMs, BHCs)	*Conduct educational meetings:* Communication-Persuasion Matrix,

	CHW role (e.g., duration in R01) and purpose/utility of MAPS+ intervention<br><br>CFIR: Intervention characteristics- relative advantage<br><br><u>Examples</u>:<br>• Staff needs upfront and ongoing reinforcement about what MAPS+ is and why it works, and training will help clarify what is expected from MAPS+ (Stakeholder meeting #1)		other organizational stakeholders, and community, patient/consumer, and family stakeholders) to teach them about the clinical innovation.	during pre- and early implementation to set clear expectations about the purpose and utility of MAPS+ intervention.<br>• Identify best practices for educating new/incoming team members about CHW-delivered MAPS+.	Social Cognitive Theory
Integrating CHW with the Team					
3	**CHW as core team member**<br><br>Integration of the CHW as a competent and valued member of the care team<br><br>CFIR: Process- planning<br><br><u>Examples</u>:<br>• With proper training with MAPS+, CHW would be a welcome member of the team because outreach is	Conduct ongoing training<br><br>Make training dynamic<br><br>Conduct educational meetings<br><br>Promote network weaving<br><br>Identify and prepare champions	*Conduct ongoing training*: Plan for and conduct training in the clinical innovation in an ongoing way.<br><br>*Conduct educational meetings*: Hold meetings targeted toward different stakeholder groups (e.g., providers, administrators, other organizational stakeholders, and community, patient/consumer, and family stakeholders) to	*Conduct ongoing training*<br>• Develop an initial orientation program for newly hired CHWs. Identify key touchpoints for ongoing training to ensure competency and continued support.<br>• Identify how existing orientation resources can be retooled for CHWs versus what material needs to be newly generated. Consider what makes CHW orientation unique from orientation for other roles.	*Conduct ongoing training*: Social Cognitive Theory<br><br>*Make training dynamic*: Social Cognitive Theory<br><br>*Conduct educational meetings*: Communication-Persuasion Matrix, Social Cognitive Theory

	so important. (Prescribing Clinician)<br>• Orientation period where they round with other members of the care team to get a sense of how things work and workflow in the clinic. (Medical Case Manager)<br>• Would be great if CHW could attend morning huddles, attend weekly meetings once they're more accustomed to them. (Medical Case Manager)<br>• The staff/clinic team is welcoming, it's helpful if the CHW is open and friendly - would be helpful to come in and meet all the staff before starting so everyone knows who MAPS CHW is and why they're there. (Medical Case Manager)<br>• Having them around as much as possible so other clinic staff recognize them and know who they are is huge, having a consistent point of contact is huge (e.g.,		teach them about the clinical innovation.<br><br>*Make training dynamic*: Vary the information delivery methods to cater to different learning styles and work contexts and shape the training in the innovation to be interactive.<br><br>*Promote network weaving*: Identify and build on existing high-quality working relationships and networks within and outside the organization, organizational units, teams, etc. to promote information sharing, collaborative problem-solving, and a shared vision/goal related to implementing the innovation.<br><br>*Identify and prepare champions*: Identify and prepare individuals who dedicate themselves to supporting, marketing, and driving through an implementation, overcoming indifference or resistance that the intervention may provoke in an organization.	• Leverage existing staff orientation processes, like shadowing with MCM. Orientation should be the same as for other staff (processes already in place).<br><br>*Make training dynamic*<br>• †In addition to MAPS+ didactics and content on HIV care, offer opportunities for CHW to round with team members, co-develop scripts for warm handoffs, engage in mock patient interactions, practice notes in Epic, and attend 1:1 meetings to develop relationships with team members.<br><br>*Conduct educational meetings*<br>• †Conduct educational meetings with the clinical team to ensure understanding of plan related to CHW onboarding, training, and integration for MAPS+ delivery.<br><br>*Promote network weaving*<br>• Building working relationships and CHW access to these relationships (via staff meetings and case conferences).<br>• Identify the extent to which organizations can leverage their working relationships to pool resources and develop shared orientation content.	*Identify and prepare champions*: Communication-Persuasion Matrix, Social Cognitive Theory, Diffusion of Innovations Theory<br><br>*Promote network weaving*: Social Network Theory

	staff meeting and viral suppression meeting attendance). (Behavioral Health Consultant)			• ┼Build on working relationships within the team to integrate CHW as a valued member. Involve CHW in both formal and informal, clinical and non-clinical team-building activities to increase cohesion (e.g., clinic retreats, skills workshops, morning huddles, coffee breaks, therapeutic debriefs, open-door or drop-by office/cubicle hours).<br>• ┼Identify key meetings that CHW should attend to increase engagement and cohesion with the team.<br><br>*Identify and prepare champions*<br>• Identify team members who dedicate themselves to supporting the CHW within the organization (e.g., advocating for CHW role, providing informal guidance related to clinic culture, serving as non-supervisory resource for CHW).	
4	**CHW presence on-site**<br><br>Physical presence of the CHW on-site and within clinics<br><br>CFIR: Inner setting-networks & communications<br><br><u>Examples</u>:	*Optimize CHW presence on-site	**Optimize CHW presence-on site*: identify key times to facilitate CHW presence at the clinic site	• ┼Identify opportunities for CHW to be present in clinic and connect with colleagues to build high-quality working relationships, information sharing, and problem-solving.<br>• ┼Identify optimal times for CHW to be present in clinic to serve as a reminder for	*Optimize CHW presence on-site*: Social Network Theory

	• Physical presence/visibility with the team. (Behavioral Health Consultant)<br>• If the person could go into the field as well as have a clinic presence, that would be beneficial. (Behavioral Health Consultant)<br>• Having a presence in the clinic so that others keep them in mind and know who/how to refer to them. (Behavioral Health Consultant)<br>• Just keep in mind that it's easier to remember to refer patients when they know the person, so meeting and letting them be part of the team and hearing from them would help. (Other)			sharing information specific to patient referrals.	
5	**Physical space constraints**<br><br>Limitations on physical space for MAPS+ delivery and/or CHW touchdown area<br><br>CFIR: Inner setting-available resources	Change physical structure and equipment<br><br>Promote network weaving	*Change physical structure and equipment*: Evaluate current configurations and adapt, as needed, the physical structure and/or equipment (e.g., changing the layout of a room, adding equipment)	*Change physical structure and equipment*<br>• Change current workspace configurations to provide physical space for both CHW to delivery MAPS+ on-site (e.g., use of exam room or empty office) and touchdown space with appropriate equipment for	*Change physical structure and equipment:* Organizational Theory (systems approach)<br><br>*Promote network weaving:* Social Network Theory

	Examples:<br>• Space is always the biggest issue, a very tiny area. (Prescribing Clinician)<br>• Space confinements- COVID and what people are comfortable with. (Administrator)<br>• Most employees are working a hybrid model right now so there are usually spaces in the clinic, would just need to plan to coordinate with other staff. (Other)		to best accommodate the targeted innovation.<br><br>*Promote network weaving*: Identify and build on existing high-quality working relationships and networks within and outside the organization, organizational units, teams, etc. to promote information sharing, collaborative problem-solving, and a shared vision/goal related to implementing the innovation.	the CHW to complete tasks (e.g., desk to write notes, computer for EHR documentation, phone for patient scheduling).<br><br>*Promote network weaving*<br>• Build on working relationships to identify opportunities for sharing workspaces and collaborative problem-solving related to space limitations.	
6	**Workflow and role clarity across the team**<br><br>Identification of scope, boundaries, and responsibilities for new CHW role in relation to other team members to promote crisp sequences of tasks within the clinic team<br><br>CFIR: Process- planning<br><br>Examples:<br>• Staff need to meet CHW to know new	Create new clinical teams<br><br>Revise professional roles<br><br>Organize clinician implementation team meetings<br><br>Promote network weaving<br><br>Conduct cyclical small tests of change<br><br>*Leverage existing processes and procedures specific to each clinic	*Create new clinical teams*: Change who serves on the clinical team, adding different disciplines and different skills to make it more likely that the clinical innovation is delivered (or is more successfully delivered).<br><br>*Revise professional roles*: Shift and revise roles among professionals who provide care, and	*Create new clinical teams*<br>• ⸆Add CHW to clinical team with clearly defined role and responsibilities specific to MAPS+ delivery and other issues that may arise in the course of patient care.<br>• Develop toolkits and pathways specific to CHW-delivered MAPS+ implementation to promote shared mental models among team members. Resources may need to further customization for each clinic's extant processes.	*Create new clinical teams:* Organizational Development Theory<br><br>*Revise professional roles:* Organizational Development Theory<br><br>*Organize clinician implementation team meetings:* Diffusion of Innovations Theory

	person has started and their role in the clinic. (Prescribing Clinician)<br>• Could be made unique compared to other roles because MCM doesn't really do the same things this person would do, more to do with concrete resource referrals, this would be unique because of the focus it would have whereas other services are broader and are dealing with multiple domains, multiple issues. (Prescribing Clinician)<br>• Not all staff would understand the MAPS CHW's role right away, would need multiple communications and explanations to make sure everyone is clear. (Other)		redesign job characteristics.<br><br>*Organize clinician implementation team meetings*: develop and support teams of clinicians who are implementing the innovation and give them protected time to reflect on the implementation effort, share lessons learned, and support one another's learning.<br><br>*Promote network weaving*: Identify and build on existing high-quality working relationships and networks within and outside the organization, organizational units, teams, etc. to promote information sharing, collaborative problem-solving, and a shared vision/goal related to implementing the innovation.<br><br>*Conduct cyclical small tests of change*: Implement changes in a cyclical fashion using small tests of change before taking changes system-wide. Tests of change benefit from	• Create a module for clinics that already have CHWs in place to distinguish new set of responsibilities specific to CHW-delivered MAPS+.<br><br>*Revise professional roles*<br>• †Shift and revise roles among team members who provide care and redesign job characteristics to avoid overlap, especially between MCM and CHW roles.<br>• †In revising roles, clearly delineate CHWs' focus on MAPS+ delivery and adherence-related issues to avoid scope creep (i.e., being assigned tasks beyond the indicated role).<br>• †Within clinical teams, identify the team member(s) who will complete key steps for MAPS+ initiation: identify whether patient meets criteria for MAPS+, initiate patient referral, discuss MAPS+ with patient, and connect patient to CHW (i.e., *who does what when*).<br>• †Identify the best approach to ensure clear communication of revised roles across all members of the team (i.e., detailed email communication, clinical flow charts or guidelines accessible on a shared drive).	*Promote network weaving:* Social Network Theory<br><br>*Conduct cyclical small tests of change*: Feedback Intervention Theory

			systematic measurement, and results of the tests of change are studied for insights on how to do better. This process continues serially over time, and refinement is added with each cycle.<br><br>**Leverage existing processes and procedures specific to each clinic:* build on extant processes and procedures within each clinic setting to integrate CHW-delivered MAPS+ into workflow	*Organize clinician implementation team meetings*<br>• Schedule regular meetings with clear agendas for key members of the team involved in MAPS+ implementation (e.g., CHW, administrator, prescribing clinician, MCM) to reflect on implementation process and troubleshoot challenges related to workflow. Consider frontloading meetings earlier in implementation with taper as processes are fine-tuned.<br><br>*Promote network weaving*<br>• Build on relationships among team members both within each clinic and across clinics with comparable processes for collaborative problem-solving to work through emergent challenges with workflow across team members and new CHW.<br><br>*Conduct cyclical small tests of change*<br>• Conduct small tests of change (i.e., 4-week duration) to evaluate changes in team roles and workflow. Refine based on pre-established measures (e.g., number of patients eligible for MAPS who participate in warm handoff for connection to CHW) and team feedback.	

				**Leverage existing processes and procedures specific to each clinic*<br>• †Many clinics have already done the work to troubleshoot and streamline; they can identify how the CHW model for MAPS+ delivery can fold into their workflow. Any changes should be simple and straightforward.	
7	**Supervision model**<br><br>Structure of supervision (both managerial and clinical) of CHW by clinic administrator and research team<br><br>CFIR: Process- planning<br><br><u>Examples</u>:<br>• Joint supervision model- assumes CHW will have accountability to someone on the research team and also would like some supervisory ability. (Administrator)<br>• Concerns: if the CHW isn't set up to succeed by having weekly supervision. There needs to be good supervision,	Provide clinical supervision<br><br>Provide local technical assistance<br><br>Provide ongoing consultation	*Provide clinical supervision*: Provide clinicians with ongoing supervision focusing on the innovation. Provide training for clinical supervisors who will supervise clinicians who provide the innovation.<br><br>*Provide ongoing consultation*: Provide ongoing consultation with one or more experts in the strategies used to support implementing the innovation.<br><br>*Provide local technical assistance*: Develop and use a system to deliver technical assistance focused on implementation issues using local personnel.	*Provide clinical supervision*<br>• †Provide timely supervision related to both managerial (i.e., schedule) and clinical (i.e., HIV care) aspects of CHW role.<br>• †Develop a joint supervision model involving both clinic administrators and research team members to ensure input from both groups of stakeholders to support the CHW's success in the role.<br><br>*Provide ongoing consultation*<br>• Provide specific consultation to the CHW related to MAPS+ delivery challenges.<br><br>*Provide local technical assistance*<br>• Provide ongoing technical assistance for the CHW specific to MAPS+ delivery (i.e., technology use like cell phone communication apps	*Provide clinical supervision:* Social Cognitive Theory<br><br>*Provide ongoing consultation:* Social Cognitive Theory<br><br>*Provide local technical assistance*: Organizational Development Theory, Diffusions of Innovation Theory

	recognition of their struggles, strengths, and limitations, needs to be able to process and ask questions, these are the concerns that caused a problem when [participant] was training peer specialists. (Behavioral Health Consultant)			shared by the team and EHR charting).	
Identifying and Referring Patients for MAPS+					
8	**Structure of existing identification and referral processes**<br><br>Existing clinic processes (i.e., Data to Care, CareWare, EHR) that identify and refer patients eligible for MAPS<br><br>CFIR: Inner setting- [n/a]<br><br><u>Examples</u>:<br>• D2C would be fine for ID, would capture the majority. (Prescribing Clinician)<br>• Referrals through CareWare, informing the provider and the doctor could inform patient. (Other)	*Leverage existing identification and referral processes<br><br>*Provider, outreach coordinator, administrator identification of patients for MAPS+ referral<br><br>Conduct educational outreach visits<br><br>Develop educational materials<br><br>Remind clinicians<br><br>Mandate change<br><br>Promote network weaving<br><br>Conduct cyclical small tests of change	**Leverage existing identification and referral processes*: use of existing clinic processes (i.e., Data to Care, CareWare, EHR) to identify and refer patients for MAPS.<br><br>**Provider, outreach coordinator, administrator identification of patients for MAPS+ referral*: team member (i.e., provider, outreach coordinator, MCM, administrator) identification of patients for MAPS+ referral to augment exclusively data-driven processes.<br><br>*Conduct educational outreach visits*: Have a	**Leverage existing identification and referral processes*<br>• ┼Use existing clinic processes (i.e., Data to Care, CareWare, EHR) to identify and refer patients for MAPS.<br><br>**Provider, outreach coordinator, administrator identification of patients for MAPS+ referral*<br>• ┼Team member (i.e., provider, outreach coordinator, MCM, administrator) identification of patients for MAPS+ referral to augment exclusively data-driven processes.<br><br>*Conduct educational outreach visits*<br>• During pre- and early implementation, study team members to conduct site	**Leverage existing identification and referral processes:* System Interaction Theory<br><br>*Provider, outreach coordinator, administrator identification of patients for MAPS referral:* System Interaction Theory<br><br>*Conduct educational outreach visits:* Social Cognitive Theory<br><br>*Develop educational materials:* Social Cognitive Theory

	• Referring would depend on the process-email or EPIC note wouldn't be a big barrier, EPIC would be very easy and probably ideal. (Prescribing Clinician)<br>• Always doing adherence checks during visits and via labs, identifying who to refer would not be a challenge- an automated system would work well, would also be easy for provider to refer. (Prescribing Clinician)<br>• Patient needs to know and agree to it before it happens- that's the only problem with automatic referral. (Prescribing Clinician)<br>• Would need to think about whether it's better to do this at the beginning, middle, or end of MCM appointment or medical appointment, think about how to integrate with the provider's appointment (for example, maybe integrate into the part of the appointment		trained person meet with providers in their practice settings to educate providers about the clinical innovation with the intent of changing the provider's practice.<br><br>*Develop educational materials:* Develop and format manuals, toolkits, and other supporting materials in ways that make it easier for stakeholders to learn about the innovation and for clinicians to learn how to deliver the clinical innovation.<br><br>*Remind clinicians*: Develop reminder systems designed to help clinicians to recall information and/or prompt them to use the clinical innovation.<br><br>*Mandate change*: Have leadership declare the priority of the innovation and their determination to have it implemented.<br><br>*Promote network weaving*: Identify and build on existing high-quality working relationships and networks within and	visits to provide education with clinical leaders (i.e., provider, outreach coordinator, administrator) in order to support the identification of eligible patients for MAPS+.<br><br>*Develop educational materials*<br>• Develop a toolkit or pathway specific to the referral process to clarify key steps and promote accountability among each team member (i.e., person X serves as "clearinghouse:" obtains updated list of potentially eligible patients, reviews list with Y, confirms eligibility with prescribing clinician, identifies when patients will present on-site for appt, and notifies CHW and prescribing clinician about potential for warm handoff. *Note*: Identify alternative plans for no shows, reschedules, walk-ins, etc.).<br>• Make the educational materials visual and easily accessible to reduce friction in the referral moment (i.e., SmartList in EHR, laminated reminder in exam room).<br><br>*Remind clinicians*<br>• With the goal of improving ease and timeliness, develop reminders for clinicians specific to (1) eligibility	*Remind clinicians:* Theories of Information Processing<br><br>*Mandate change:* Behavioral Economic Theory<br><br>*Promote network weaving:* Social Network Theory<br><br>*Conduct cyclical small tests of change:* Feedback Intervention Theory

	when they're discussing labs). (Administrator)<br>• Would also want to have provider be able to ID.<br>• Would want to be able to refer individuals outside of just D2C- might be helpful to run referrals through weekly meetings. (Prescribing Clinician)<br>• Already use D2C, would be easy to print a report and share it with the CHW; want to make sure to loop in [administrator] and Outreach Coordinator to decide if CHW should reach out to particular client- would want to be able to also refer by provider/outside of D2C. (Administrator)<br>• Provider needs to know the criteria for referral-they have so many things that they're trying to juggle, short visits with patients, so who/when/how to refer for MAPS need to be clear and easy. (Behavioral Health Consultant)		outside the organization, organizational units, teams, etc. to promote information sharing, collaborative problem-solving, and a shared vision/goal related to implementing the innovation.<br><br>*Conduct cyclical small tests of change*: Implement changes in a cyclical fashion using small tests of change before taking changes system-wide. Tests of change benefit from systematic measurement, and results of the tests of change are studied for insights on how to do better. This process continues serially over time, and refinement is added with each cycle.	criteria and (2) actual act of referring to encourage participation in MAPS+ implementation.<br>• †Bake time into established meetings to review automated (i.e., data generated) referrals as an engagement reminder.<br><br>*Mandate change*<br>• Mandate team member participation in MAPS+ referral process if engagement is poor.<br><br>*Promote network weaving*<br>• Build on working relationships within the clinic to engage in problem-solving for any identification or referral challenges (e.g., if Data to Care list is not up to date and needs to be cross-checked with EHR- who will update, how frequently, optimal processes across key team members)<br><br>*Conduct cyclical small tests of change*<br>• Conduct small tests of change (i.e., 4-week duration) to evaluate workflow procedures specific to patient identification and referral using existing systems (i.e., Data to Care, CareWare, Epic). Refine based on pre-established	

				measures (e.g., number of patients identified in CareWare as eligible for MAPS but ineligible per more updated EHR) and team feedback.	
Connecting Patients and CHWs					
9	**CHW scheduling and availability**<br><br>CHW work schedule and accessibility for patients and team members<br><br>CFIR: Process- planning<br><br>Examples:<br>• Would want to make sure CHW available to attend appointments the patient was having with this clinician. (Prescribing clinician)<br>• Helpful to be available by after hours and via text message. (Stakeholder Meeting #1)<br>• What happens if patients walk in on a day that CHWs are not on-site? (Stakeholder Meeting #1)<br>• Need to be able to get in touch with them	*Match scheduling to clinic needs<br><br>Conduct cyclical small tests of change	**Match scheduling to clinic needs:* collect and analyze data related to the need for the innovation.<br><br>*Conduct cyclical small tests of change*: Implement changes in a cyclical fashion using small tests of change before taking changes system-wide. Tests of change benefit from systematic measurement, and results of the tests of change are studied for insights on how to do better. This process continues serially over time, and refinement is added with each cycle.	**Match scheduling to clinic needs*<br>• Identify top blocks of time related to patient volume/needs during within each clinic for which CHW should be available to attend appointments, participate in warm handoffs, and/or connect about patient care via phone/email/text<br><br>*Conduct cyclical small tests of change*<br>• Conduct small tests of change (i.e., 4-week duration) to evaluate benefits and burdens of specific schedules and accessibility of the CHW. Make refinements as needed (i.e., CHW clinic attendance on Monday mornings vs. Tuesday afternoons may work better for site depending on patient volume and prescribing clinician schedules; same day phone calls with CHW referrals may be less efficient than 2x/week batched calls).	**Match scheduling to clinic needs:* System Interaction Theory<br><br>*Conduct cyclical small tests of change:* Feedback Intervention Theory

	in an accessible and flexible fashion. (Other)			• †Clarify team expectations of CHW accessibility as CHW role is fine-tuned.	
10	**Initial contact between CHW and patient**<br><br>First connection between patients and CHWs<br><br>CFIR: Process- planning<br><br><u>Examples</u>:<br>• Would love for CHWs to come into clinic for warm handoff. (Prescribing Clinician)<br>• Lots of patients are sensitive to inviting new people into their care, would need to use baby steps and a warm handoff, ask how patient feels about inviting new person in. (Administrator)<br>• Staff member (e.g., MCM) introduce CHW and patient to review goals and establish trust, help them feel comfortable - affirmation from a trusted source. (Stakeholder Meeting #1)<br>• CHW in the room with the clinician and client	*Warm handoff	*Warm handoff:* transparent transfer of care between two members of the health care team, where the handoff occurs in front of the patient	*Warm handoff*<br>• †Enactment of MAPS+ referral in front of/with the patient, in which a team member with an established relationship with the patient connects him/her/them to the CHW, explaining why the CHW can address adherence challenges and emphasizing the CHW's competence.<br>• †Increase comfort and ease of communication by offering patients business cards with CHW phone number and photo and/or invite patients to save CHW's work cell phone number in their phones before leaving the clinic site.<br>• Develop checklists or job aids specific to warm handoff procedures and language to ensure consistent messaging across patients.<br>• Conduct warm handoff practice sessions to evaluate changes in workflow that may occur with 2 team members present.<br>• Warm handoffs to CHWs would blend in well with what clinic already does. Form will look different across clinics. Even brief introductions are	*Warm handoff*: Social Support Theory

	(at least to start) to demonstrate clinician buy-in and CHW as part of the team - introduction invests CHW with clinician's authority. (Stakeholder Meeting #1)			positive (name + face). Good handoffs are tied into an understanding of others' roles.	
11	**CHW characteristics**<br><br>Characteristics of the CHW (e.g., demographics, experiences, attitudes, skills)<br><br>CFIR: Characteristics of individuals involved- [n/a]<br><br><u>Examples</u>:<br>• Team member who reflects the population that we serve; someone positive and calming. (Prescribing Clinician)<br>• Good balance between working independently and being able to put out fires on their own and knowing when to ask for help; cultural competence is huge; communication skills; sense of boundaries- you need to have a good sense of	Conduct ongoing training<br><br>Involve patients/consumers and family members	*Conduct ongoing training*: Plan for and conduct training in the clinical innovation in an ongoing way.<br><br>*Involve patients/consumers and family members*: Engage or include patients/consumers and families in the implementation effort.	*Conduct ongoing training*<br>• ┼Provide ongoing training for the CHW not just related to MAPS+ but also ethical implications of self-disclosure, boundary setting, duality of role, cultural responsiveness, and trauma-informed care.<br><br>*Involve patients/consumers and family members*<br>• Elicit feedback from patients on the qualities they seek in a CHW team member.<br>• Identify opportunities in the course of care for patients to provide feedback (both benefits and burdens) on the CHW interventionist model for MAPS+ delivery.	*Conduct ongoing training*: Social Cognitive Theory, Social Support Theory, Theories of Stigma and Discrimination<br><br>*Involve patients/consumers and family members:* Person-Centered Care Model

	personal safety and responsibility. (Behavioral Health Consultant)				
12	**Individual-level HIV-related stigma**<br><br>Micro-level stigma (experienced, perceived, anticipated, or internalized) that has the potential to influence HIV-related health behaviors of PWH, specifically CHW-delivered MAPS+ engagement<br><br>CFIR: Inner setting- [n/a]<br><br>Examples:<br>• Stigma factor has to be thought about, not usually a self-reported barrier but is a problem. (Policymaker)<br>• Thinks MAPS+ would be very helpful for reducing disparities for reasons mentioned before, ability to share experience with stigma serves a large role in establishing trust with patients, making them feel welcome and	Facilitation<br><br>Involve patients/consumers and family members<br><br>Conduct local needs assessment	*Facilitation*: A process of interactive problem solving and support that occurs in a context of a recognized need for improvement and a supportive interpersonal relationship.<br><br>*Involve patients/consumers and family members*: Engage or include patients/consumers and families in the implementation effort.<br><br>**Identify local approaches to relationship building:* identify strategies that clinics use in routine care to build trust and rapport with patients.	*Facilitation*<br>• Provide implementation facilitation to support CHWs' connection to patients and address more nuanced challenges with engagement.<br><br>*Involve patients/consumers and family members*<br>• Elicit feedback from patients about preferences for linkage to CHW, meeting locations (i.e., clinic, local community, virtual), etc.<br>• Identify strategies for CHW to appropriately navigate and communicate insider-outsider-intermediary status.<br>• Identify opportunities in the course of care for patients to provide feedback (both benefits and burdens) on the CHW interventionist model for MAPS+ delivery.<br><br>**Identify local approaches to relationship building:*<br>• Identify and share relational strategies that clinics use to mitigate stigma and establish relationships with patients in order to optimize care delivery.	*Facilitation:* Social Cognitive Theory<br><br>*Involve patients/consumers and family members*: Person-Centered Care Model<br><br>*Identify local approaches to relationship-building:* Theories of Stigma and Discrimination, Diffusion of Innovations Theory

	comfortable. (Behavioral Health Consultant)<br>• The CHW will be able to "use parts of themselves to help others" which will help with shame and stigma, speed up process of gaining trust. (Behavioral Health Consultant)<br>• Flexibility of having someone in the field, might help get around stigma. (Medical Case Manager)				
Delivering MAPS+					
13	**MAPS+ characteristics**<br><br>Components and features of MAPS+ intervention<br><br>CFIR: Intervention characteristics-adaptability<br><br><u>Examples</u>:<br>• More structured and time-limited than current strategies. (Prescribing Clinician)<br>• Likes that this provides a more structured way of approaching adherence	Promote adaptability	*Promote adaptability*: Identify the ways a clinical innovation can be tailored to meet local needs and clarify which elements of the innovation must be maintained to preserve fidelity.	*Promote adaptability*<br>• †Identify the ways in which MAPS+ characteristics can be tailored to meet the needs of local clinics and patients while maintaining core components. For example, per stakeholder feedback, consider decreasing the length or number of sessions (if intervention effectiveness can be maintained).<br>• †Leverage the flexibility of MAPS+ delivery to offer multiple options for MAPS+ delivery specific to timing (i.e., before/after 9a-5p work hours, on weekends), location (in clinic versus in the community), and method	*Promote adaptability:* Diffusion of Innovations Theory

	challenges, very proceduralized and easy to track what has happened in terms of care received. (Prescribing Clinician)<br>• Loves that patients make their own goals/have autonomy over their own care. (Administrator)<br>• Need to be accessible to clients with a variety of education levels and comfort levels in English.			(in-person versus virtual) to best meet patients' needs and preferences.	
14	**Patients' perceptions of CHW-delivered MAPS+**<br><br>Perceived burdens/costs or benefits conferred by MAPS engagement<br><br>CFIR: Intervention characteristics-complexity<br><br><u>Examples</u>:<br>• There will always be patients who aren't on board, won't want to engage with a new person, added patient burden. (Other)	Distribute educational materials<br><br>Obtain and use patients/consumers and family feedback<br><br>Promote network weaving	*Distribute educational materials*: Distribute educational materials (including guidelines, manuals, and toolkits) in person, by mail, and/or electronically.<br><br>*Promote network weaving*: Identify and build on existing high-quality working relationships and networks within and outside the organization, organizational units, teams, etc. to promote information sharing, collaborative problem-solving, and a shared vision/goal related to	*Distribute educational materials*<br>• Distribute educational/promotional materials to clinic team and patients with specific messaging about MAPS+ as an intervention geared toward shared effort and collaboration to improve adherence. Ensure that team is consistent in this messaging to patients as well in order to avoid mixed signals.<br><br>*Promote network weaving*<br>• Build on working relationships to ensure good communication between team members with the goal of avoiding inappropriate	*Distribute educational materials*: Communication-Persuasion Matrix, Social Cognitive Theory<br><br>*Promote network weaving*: Social Network Theory<br><br>*Obtain and use patients/consumers and family feedback:* Person-Centered Care Model

	• Potential issues could include fidelity to the intervention and issues with time (patient burden). (Prescribing Clinician)<br>• 60-90 minutes is way, way too long because of burden on patient; we don't want to make folks feel like we're putting more work on them. (Policymaker)<br>• MAPS framed as shared burden of work to help get patient to successful adherence and retention important so they don't feel overwhelmed. (Stakeholder Meeting #1)		implementing the innovation.<br><br>*Obtain and use patients/consumers and family feedback*: Develop strategies to increase patient/consumer and family feedback on the implementation effort.	overlap/duplication of services that would create additional burden for patients. Note distinction: brief reinforcement of adherence strategies by MCM, for example, may be clinically indicated.<br><br>*Obtain and use patients/consumers and family feedback*<br>• Routinely elicit feedback from patients as key end users – is CHW-delivered MAPS+ acceptable and if not, what aspects can be tailored to facilitate ongoing patient engagement?	
Coordinating Care Between CHW and Team					
15	**Care coordination**<br><br>Communication between CHW and team members about MAPS+ engagement<br><br>CFIR: Inner setting- networks & communications	Change record systems<br><br>Centralize technical assistance<br><br>Organize clinician implementation team meetings<br><br>Promote network weaving	*Change record systems*: Change records systems to allow better assessment of implementation or clinical outcomes.<br><br>*Centralize technical assistance*: Develop and use a centralized system to deliver technical	*Change record systems*<br>• Identify clinics' preferences for EHR-based communication (i.e., notes in chart, messaging).<br>• †Create templates in Epic for CHWs that are tailored to each clinic's communication preferences. Ensure that templates are easy and	*Change record systems:* Organizational Development Theory<br><br>*Centralize technical assistance:* Organizational Development Theory

	<u>Examples</u>:<br>• CHWs create a very condensed document like a treatment plan (4 points) to communicate goals with provider and other staff (easier reference than searching through notes). (Stakeholder Meeting #1)<br>• It would be critical to have a system that would allow CHW to communicate with MCMs for individuals who have MCMs, as well as a system for patients who don't have MCMs. Ideal would be to have a platform where everyone can use centralized, streamlined communication. Already meeting-heavy, weekly meeting probs not the best idea -- using the chart for specific communications (e.g., this individual needs to be called this week) would be best. (Other)<br>• Ideally if they have access to Epic, if they could write notes	*Research team presentation at community-based organization meeting	assistance focused on implementation issues.<br><br>*Organize clinician implementation team meetings*: Develop and support teams of clinicians who are implementing the innovation and give them protected time to reflect on the implementation effort, share lessons learned, and support one another's learning.<br><br>*Promote network weaving*: Identify and build on existing high-quality working relationships and networks within and outside the organization, organizational units, teams, etc. to promote information sharing, collaborative problem-solving, and a shared vision/goal related to implementing the innovation.<br><br>**Research team presentation at community-based organization meeting*: research team presentation to communication to community-based	efficient for CHW to use and team members to read.<br>• †Identify non-EHR communication systems, like bulleted treatment plans, to increase communication between the CHW and care team, as an alternative or augment to EHR documentation.<br><br>*Centralize technical assistance*<br>• Use a centralized system for delivering technical assistance for EHR use.<br><br>*Organize clinician implementation team meetings*<br>• Schedule regular meetings with clear agendas for key members of the team involved in MAPS+ implementation (e.g., CHW, administrator, prescribing clinician, MCM) to reflect on implementation process and identify opportunities to improve communication.<br>• †Leverage existing meetings, like QI or weekly meetings, for MAPS+-related communication.<br><br>*Promote network weaving*<br>• †Clarify who needs what information within the clinical team and ensure that relationships are in place for this information exchange	*Organize clinician implementation team meetings:* Organizational Development Theory<br><br>*Promote network weaving:* Social Network Theory<br><br>*Research team presentation at community-based organization meeting*: Social Network Theory

	based on communication and cc this participant and the provider, is also the opportunity for an ask like need refill etc. (Prescribing Clinician)<br>• EMR is “bible” and central for communication. (Stakeholder Meeting #1)<br>• No problems with workflow if it's in EPIC, providers send messages to one another that way but not all providers read notes. (Stakeholder Meeting #1)		organizations that provide medical case management for PWH	(i.e., between prescribing clinicians and CHW).<br>• Build on working relationships within the team to promote information sharing and leverage existing processes, like group text messages, to facilitate communication.<br>• Identify successful communication strategies used in other clinics and consider trials within home clinic site to support CHW-delivered MAPS+.<br><br>**Research team presentation at community-based organization meeting*: research team presentation to communication to community-based organizations that provide medical case management for PWH	
16	**CHW knowledge of cross-clinic processes**<br><br>CHW knowledge of unique processes/procedures across assigned clinics<br><br>CFIR: Inner setting-networks & communications<br><br><u>Examples</u>:<br>• Determinant generated through research team small	Conduct ongoing training<br><br>Remind clinicians	*Conduct ongoing training*: Plan for and conduct training in the clinical innovation in an ongoing way.<br><br>*Remind clinicians*: Develop reminder systems designed to help clinicians to recall information and/or prompt them to use the clinical innovation.	*Conduct ongoing training*<br>• Conduct ongoing training for CHWs about processes specific to their assigned clinics.<br><br>*Remind clinicians*<br>• Create reminders (e.g., tip sheets) about specific clinic processes to support CHW retention and understanding of key information.	*Conduct ongoing training:* Social Cognitive Theory<br><br>*Remind clinicians:* Theories of Information Processing

	group discussions based on interpretation of interview data and recognized need for CHWs to be knowledgeable about clinic processes to which he/she/they are assigned (as CHWs will be assigned to 2 clinics in the R01)				
Sustaining MAPS Implementation					
17	**Ongoing team buy-in**<br><br>Continued buy-in and engagement from the care team specific to CHW-delivered MAPS<br><br>CFIR: Characteristics of individuals- knowledge & beliefs about the intervention<br><br><u>Examples</u>:<br>• Celebrate successes, produce data about how well (and if) the strategy is working; any way to demonstrate effectiveness is helpful; being able to show outcomes (e.g., we linked 17 ppl to management this month). (Other)	Develop and implement tools for quality monitoring<br><br>Facilitate relay of clinical data to providers	*Develop and implement tools for quality monitoring*: Develop, test, and introduce into quality-monitoring systems the right input—the appropriate language, protocols, algorithms, standards, and measures (of processes, patient/consumer outcomes, and implementation outcomes) that are often specific to the innovation being implemented.<br><br>*Facilitate relay of clinical data to providers*: Provide as close to real-time data as possible about key measures of process/outcomes using integrated	*Develop and implement tools for quality monitoring*<br>• †Clinic teams receive numerous data reports. CHW-related outcomes should be spotlighted to increase both attention and buy-in from the team. QI is most meaningful when it is disaggregated and patient-centered: keep the focus on patient success.<br>• Identify owner of quality monitoring process.<br>• Identify key measures and outcomes for MAPS+ quality monitoring (e.g., number of patients identified for referral, proportion of referrals initiated and connected to CHW, number of MAPS sessions/patient completed, percent improvement in adherence).	*Develop and implement tools for quality monitoring:* Feedback Intervention Theory<br><br>*Facilitate relay of clinical data to providers:* Feedback Intervention Theory

	• QI meeting, maybe at the 6-month mark share some metrics about how MAPS is helping patients, demonstrate that through your (the team) help, we've executed x# of MAPS sessions with x# of patients, if you can show how the viral load has been impacted for 5-10 MAPS people, and if patients have improved, the team knows they've helped make that happen by facilitating MAPS CHW work. (Policymaker)		modes/channels of communication in a way that promotes use of the targeted innovation.	• Identify frequency of data collection.<br>• Identify best forum for clinic-specific feedback: QI meeting, weekly meetings, etc.<br>• †Celebrate success – customize celebrations specific to each clinic. Publicly acknowledge individual team member's contributions to MAPS+' effectiveness.<br><br>*Facilitate relay of clinical data to providers*<br>• †Ensure that prescribing clinicians, whose schedules may preclude participation in QI and/or weekly meetings, receive timely outcome data to promote their continued engagement.<br>• Link data output to CHW-related outcomes to increase investment by the team.	
Sociopolitical context					
18	**Structural stigma**<br><br>Societal-level conditions, cultural norms, and institutional practices that constrain opportunities, resources, and wellbeing<br><br>CFIR: Outer setting-[n/a]	Conduct ongoing training<br><br>Provide ongoing consultation	*Conduct ongoing training*: Plan for and conduct training in the clinical innovation in an ongoing way.<br><br>*Provide ongoing consultation*: Provide ongoing consultation with one or more experts in the strategies used to	*Conduct ongoing training*<br>• Conduct ongoing training for CHWs on how structural stigma affects patients (particularly how stigma related to HIV may be compounded by stigma associated with poverty, substance use, and sexual minority status) and may influence MAPS+ implementation. Consider	*Conduct ongoing training:* Social Cognitive Theory, Theories of Stigma and Discrimination<br><br>*Provide ongoing consultation:* Social Cognitive Theory, Theories of Stigma and Discrimination

	<u>Example</u>:<br>• Many of these young Black men who were diagnosed with HIV within the last few years, this is truly their first time really interacting with the healthcare system. And because of that, no one's ever expressed to them what the healthcare system is, and what it can do for you, and what your rights are as a patient. (Administrator)<br>• Problems with lab; often people at the lab won't see our patients. (Administrator)<br>• Clinic location as a barrier (i.e., parking) but benefit of anonymity ("less likelihood of being outed as positive"). (Policymaker)		support implementing the innovation.	including other conceptual frameworks on HIV-related stigma and adherence.<br>• Training can optimize ways to support patients (e.g., meeting patients in their preferred locations, referring for to best resource to navigate specific challenges- like labs or insurance).<br><br>*Provide ongoing consultation*<br>• Provide ongoing consultation for CHWs on managing challenging situations equitably (e.g., no shows for sessions).	
19	**Structural racism**<br><br>A system in which public policies, institutional practices, cultural representations, and other norms work in various, often reinforcing ways to perpetuate racial group inequity	Conduct ongoing training<br><br>Provide ongoing consultation	*Conduct ongoing training*: Plan for and conduct training in the clinical innovation in an ongoing way.<br><br>*Provide ongoing consultation*: Provide ongoing consultation with one or more experts in	*Conduct ongoing training*<br>• Provide training for CHWs regarding the ways in which manifestation of structural racism may impact patients, influence MAPS uptake, and opportunities for advocacy within their organization and/or community (e.g., potential	*Conduct ongoing training:* Social Cognitive Theory, Theories of Stigma and Discrimination<br><br>*Provide ongoing consultation:* Social Cognitive Theory,

	CFIR: Outer setting- [n/a]<br><br>Example:<br>• Structural racism: whether providers believe someone will adhere can impact the way they interact with patient, can impact what kind of resources and communication they pass along to patients; poverty is statistically linked with race which can have an impact; prescriber implicit bias; some sites require appointments, if you no-show can be billed, that's enough to keep some patients from trying to stay in treatment- people with money and good insurance can bypass those barriers. (Policymaker)<br>• 2020 systematic racism: structural barriers related to historical and institutional racism, structures are in place to discriminate. (Policymaker)		the strategies used to support implementing the innovation.	patient experiences of finding health care settings unreceptive; medical distrust rooted in historical context of past medical exploitation).<br><br>*Provide ongoing consultation*<br>• Provide ongoing consultation for specific challenges related to MAPS implementation in the context of structural racism (e.g., link between area deprivation index and lower likelihood of HIV viral suppression)	Theories of Stigma and Discrimination

	• Do think this [structural racism] contributes to patient ability to adhere/stay retained: insurance can be difficult to navigate, policy changes to social security or Medicare, prescription cost can get in the way of getting meds, at this clinic they accept folks sans insurance, but specialists often don't, so cost can be a problem. (Medical Case Manager)				
20	**Poverty**<br>Lack of financial resources and essentials for a minimum standard of living<br><br>CFIR: Outer setting-patient needs & resources<br><br><u>Examples</u>:<br>• In Philly, see a higher incidence of disparity at the interrelation of institutional racism, severe poverty. Philadelphia is a majority minority population- most extremely poor	Conduct ongoing training<br><br>Provide ongoing consultation<br><br>Involve patients/consumers and family members<br><br>*Communicate feedback on structural barriers back to clinic leadership and PDPH	*Conduct ongoing training*: Plan for and conduct training in the clinical innovation in an ongoing way.<br><br>*Provide ongoing consultation*: Provide ongoing consultation with one or more experts in the strategies used to support implementing the innovation.<br><br>*Involve patients/consumers and family members*: Engage or include patients/consumers and families in the implementation effort.	*Conduct ongoing training*<br>• Conduct ongoing training for CHWs on the landscape of poverty in Philadelphia, trends in the needs of patients served, and key community resources.<br><br>*Provide ongoing consultation*<br>• Provide specific consultation for CHWs encountering limited resources as a key adherence-related challenge for their patients (i.e., patient-specific resources based on the situation). Identify role of MCM in consultation.	*Conduct ongoing training:* Social Cognitive Theory, Theories of Stigma and Discrimination<br><br>*Provide ongoing consultation:* Social Cognitive Theory, Theories of Stigma and Discrimination<br><br>*Involve patients/consumers and family members*: Person-Centered Care Model

	neighborhoods are Black neighborhoods, disproportionate burden of disease broadly (diabetes, COVID, HIV), and that creates real barriers; need to also keep in mind the resilience and resistance that you see in these communities. (Policymaker)		*Communicate feedback on structural barriers back to clinic leadership and PDPH*: Ensure that CHWs have a mechanism for collecting information on structural barriers and communicating this back to PDPH.	*Involve patients/consumers and family members*<br>• Explicitly engage patients in ongoing communication related to their specific needs and how resources impact engagement with CHWs and MAPS+.<br><br>*Communicate feedback on structural barriers back to clinic leadership and PDPH*<br>• Develop pathway with timed checkpoints for CHWs to update leadership on challenges experienced by patients that impact MAPS+ participation.	
21	**Intersectional marginalization**<br><br>Experiences of discrimination and systemic exclusion due to the specific intersection of individuals' multiple identities<br><br>CFIR: Outer setting- [n/a]<br><br><u>Examples</u>:<br>• Young gay people who already face shame/stigma for coming out; stigma compounded by	Conduct ongoing training<br>Provide ongoing consultation	*Conduct ongoing training*: Plan for and conduct training in the clinical innovation in an ongoing way.<br><br>*Provide ongoing consultation*: Provide ongoing consultation with one or more experts in the strategies used to support implementing the innovation.	*Conduct ongoing training*<br>• Conduct ongoing training for CHWs on the concurrent, multiple, and embedded social locations that may influence experiences of PWH.<br><br>*Provide ongoing consultation*<br>• Provide case-specific consultation to CHWs on challenges encountered in MAPS+ delivery specific to patients' experiences with intersectional marginalization and exclusion.	*Conduct ongoing training:* Social Cognitive Theory, Theories of Stigma and Discrimination<br><br>*Provide ongoing consultation:* Social Cognitive Theory, Theories of Stigma and Discrimination

	HIV+ status. (Behavioral Health Consultant)<br>• Understanding for whatever reason what it feels like to live with stigma, again, would be a huge thing, which is really hard to interview on because stigma can come from many things that are not just HIV. Again, you're LGBTQ status, but can also be many other things. (Administrator)<br>• HIV disparities impact Black and Latinx communities, see disparities magnified across intersectional populations- risk groups that have been identified including people who inject drugs, trans folks, LGBTQ+ community all disproportionately impacted. (Policymaker)				
22	**Medical hierarchy**	Conduct ongoing training	*Conduct ongoing training*: Plan for and conduct training in the	*Conduct ongoing training*<br>• Provide training for CHWs for navigating the health	*Conduct ongoing training:* Social Cognitive Theory

	Systems of power, privilege, and authority in health care that may manifest with prescribing clinicians having higher status<br><br>CFIR: Outer setting- [n/a]<br><br><u>Examples</u>:<br>• Having doctor buy-in is massively important-they need to not ignore the flag in the EMR, this needs to be front of their mind and they need to be willing to engage with it. (Policymaker)<br>• Ability to speak "up and down the medical hierarchy" and "up and down the SES ladder." (Behavioral Health Consultant)<br>• Could be a trust level in terms of the hierarchy in the clinic (providers could consider auxiliary staff to be less trustworthy, may create a barrier to developing the trust required to refer someone and pass along that part of their care). (Policymaker)	Provide ongoing consultation<br><br>Facilitation	clinical innovation in an ongoing way.<br><br>*Provide ongoing consultation*: Provide ongoing consultation with one or more experts in the strategies used to support implementing the innovation.<br><br>*Facilitation*: A process of interactive problem solving and support that occurs in a context of a recognized need for improvement and a supportive interpersonal relationship.	care system, communication within each clinic, and challenges that patients may perceive with communication specific to MAPS+/adherence/retention (e.g., perceived privilege of medical expertise over patients' knowledge/experience).<br><br>*Provide ongoing consultation*<br>• Provide consultation for CHWs if challenges specific to communication within the medical team arise.<br><br>*Facilitation*<br>• Use facilitation to identify challenges specific to clinic-specific medical hierarchies and opportunities to navigate in order to support CHW-delivered MAPS+.	*Provide ongoing consultation:* Social Cognitive Theory<br><br>*Facilitation*: Social Cognitive Theory

	• "Good job for thinking about people from the community" because the system is so highly professionalized and we often forget about how much community members have to offer even if they don't have formal training or a college degree. (Policymaker)				
23	**Representation of shared identity**<br><br>Identification of common qualities/characteristics across clinic staff and patients<br><br>CFIR: Outer setting-[n/a]<br><br><u>Examples</u>:<br>• We don't know how to do adherence that well for people who are different from us [the providers, broadly]. The more different from us they are-lower SES, less education, different lived experience, the harder it is for us to get it right when thinking about the	Conduct ongoing training<br><br>Provide ongoing consultation	*Conduct ongoing training*: Plan for and conduct training in the clinical innovation in an ongoing way.<br><br>*Provide ongoing consultation*: Provide ongoing consultation with one or more experts in the strategies used to support implementing the innovation.	*Conduct ongoing training*<br>• Provide training for CHWs on the patient population served, as well as appropriate boundaries related to self-disclosure and use of self as therapeutic tool.<br><br>*Provide ongoing consultation*<br>• Provide case-specific consultation on issues that may arise as the CHW navigates commonalities in lived experiences with patients.	*Conduct ongoing training:* Social Cognitive Theory<br><br>*Provide ongoing consultation:* Social Cognitive Theory

	motivators and barriers for them. (Administrator)<br>• Medical mistrust in the African American community can be a problem; almost all providers are white, the people of color who do work in this clinic seem to be really meaningful to patients. (Behavioral Health Consultant)				
24	**Tech disparities**<br><br>Differences in access to technology (e.g., phone, internet) that impact treatment<br><br>CFIR: Outer setting-patient needs & resources<br><br>Examples:<br>• No cell phone (particularly in AA population served). (Prescribing Clinician)<br>• Real racial disparity in telemedicine- If you don't have access to right tech, telemed isn't a viable option for you. (Prescribing Clinician)<br>• Difficult contacting and locating patients-	Involve patients/consumers and family members	*Involve patients/consumers and family members*: Engage or include patients/consumers and families in the implementation effort.	*Involve patients/consumers and family members*<br>• CHW to engage with patients to determine optimal approaches for communication in light of differential access to technology and brainstorm strategies to promote access to CHW-delivered MAPS+. CHW to coordinate with MCM regarding previously trialed resources (e.g., prepaid call cards).	*Involve patients/consumers and family members:* Person-Centered Care Model

	Concern for a similar pattern playing out in which patients do not connect due to instability, homelessness (shelters/hospital), no phone. (Stakeholder Meeting #1)				
25	**Comprehensive social services**<br><br>Referral and linkage to valuable social services, including medical insurance, housing, utility payments, food, and mental health therapy<br><br>CFIR: Outer setting-patient needs & resources<br><br><u>Examples</u>:<br>• Making sure patients have medical insurance, making sure their needs are met to allow them to meet HIV care goals (e.g., transport, housing, clothing, legal); helping them navigate the system.	Promote network weaving	*Promote network weaving*: Identify and build on existing high-quality working relationships and networks within and outside the organization, organizational units, teams, etc. to promote information sharing, collaborative problem-solving, and a shared vision/goal related to implementing the innovation.	*Promote network weaving*<br>Identify and build upon working relationships within networks within the city and region to promote information-sharing and problem-solving related to social service access for patients experiencing challenges related to food insecurity, housing, etc.	*Promote network weaving*: Social Network Theory
26	**Norms of dignity and respect in HIV care**	Promote network weaving	*Promote network weaving*: Identify and build on existing high-	*Promote network weaving*<br>• Build on working relationships within and	*Promote network weaving:* Social Network Theory

	Sensitivity, responsivity, and respect deeply embedded within HIV care and treatment<br><br>CFIR: Outer setting- [n/a]<br><br><u>Examples</u>:<br>• Impressed with the treatment of patients in the clinic, large emphasis on treating patients with respect, dignity, ensuring that they feel cared for, being mindful of stigma. (Behavioral Health Consultant)<br>• [Clinic environment] tries to address it, open convo about race, don't want to be judgmental; telling clients that their lived experience is valued and that staff is honored that clients will share experiences with us; see one another as family. (Administrator)<br>• Environment: very supportive, try to make patients feel safe, feel like family, feel supported; try to build a bridge with the community, e.g. with		quality working relationships and networks within and outside the organization, organizational units, teams, etc. to promote information sharing, collaborative problem-solving, and a shared vision/goal related to implementing the innovation.	across clinics in the city and region to share strategies that support culture of respect and dignity within care delivery (e.g., development of community bridges through Covid vaccination programming)	

	COVID vaccine.<br>(Other)				
	Additional strategies derived from Stakeholder Meeting #2:<br><br>**Integrate research team into learning collaboratives:* research team engagement with PDPH AIDS Activities Coordinating Office learning collaborative that fosters communication about new initiatives<br><br>**Research team engagement with a collaborative between HIV care and prevention service users and providers:* research team engagement with a collaborative of public health stakeholders, including HIV care and prevention service users and providers, to facilitate MAPS+ sustainability				
